# Lipid Nanoparticles for Gene Therapy: Unresolved Challenges in Manufacturing, Transdermal Delivery, Machine Learning, Endosomal Escape, and the Protein Corona

**DOI:** 10.3390/pharmaceutics18080991

**Published:** 2026-08-11

**Authors:** Ognjen Milić, Sanela M. Savić, Melanija Zurković, Boban Stanojević, Snežana Savić

**Affiliations:** 1 Department of Pharmaceutical Technology and Cosmetology, Faculty of Pharmacy, University of Belgrade, 11221 Belgrade, Serbia; ognjen.milic@pharmacy.bg.ac.rs (O.M.); melanija.zurkovic@pharmacy.bg.ac.rs (M.Z.); 2 Faculty of Technology in Leskovac, University of Niš, 16000 Leskovac, Serbia; 3 R&D Sector, DCP Hemigal, 16000 Leskovac, Serbia; 4 Department of Pharmacology, Faculty of Pharmacy, University of Belgrade, 11221 Belgrade, Serbia; boban.stanojevic@pharmacy.bg.ac.rs

**Keywords:** lipid nanoparticles, mRNA delivery, ionizable lipids, microfluidics, machine learning, endosomal escape, microneedle, formulation optimization

## Abstract

Lipid nanoparticles (LNPs) are now the leading delivery platform for nucleic acid therapeutics, but progress in the field is measured almost entirely by physicochemical and computational proxies rather than by functional properties that determine therapeutic outcomes. This review examines six interconnected areas of LNP development: microfluidic manufacturing, lyophilization, transdermal microneedle delivery, machine learning-guided formulation design, endosomal escape biology, and protein corona-mediated organ targeting. Although these areas are often discussed separately, they are linked by a common gap between routinely measured physicochemical or computational endpoints and the biological outcomes that determine therapeutic performance. A recently developed antifouling coating substantially reduced microfluidic channel fouling under the tested conditions, although its scalability remains to be validated. Lyophilization, by contrast, still requires formulation specific re-optimization for each new lipid composition, which remains an important barrier to clinical translation. In microneedle-based delivery, physicochemical integrity after fabrication is routinely treated as a proxy for therapeutic function, although, to our knowledge, no published study has directly compared endosomal escape capacity before and after microneedle fabrication. In machine learning, model accuracy is limited primarily by fragmented, outcome-biased training data rather than by algorithm design. Independent measurements of endosomal escape efficiency converge on a low ceiling whose biological origin, whether lipid-specific or inherent to the mechanism, remains unknown. For organ-selective targeting, one mechanistic account rests on a hypothesis tested in advance; another, equally prominent, has not been shown to have been anticipated rather than reconstructed after the fact. Closing this gap is now the field’s central methodologically priority.

## 1. Introduction

### 1.1. From Parenteral Nutrition to Gene Therapy

Lipid-based delivery systems have been used in modern medicine since the development of intravenous emulsions for total parenteral nutrition in the 1960s, when soybean oil and egg lecithin were first established as safe components for intravenous administration [1]. Over the next 50 years, new lipid emulsion compositions were systematically developed and evaluated to improve safety and efficacy, but challenges in physical stability, compatibility, and clinical optimization persist [2,3]. In parallel, the same physicochemical principles were applied to drug delivery, leading to commercial drug-loaded parenteral emulsions such as Diazemuls and Diprivan [1]. Characterization frameworks and stability testing protocols originally developed for parenteral nutrition emulsions, including measurements of droplet size, zeta potential (ZP), pH, and osmolality, remain fundamental to modern lipid-based drug delivery systems [1], as demonstrated in the development of polyethylene glycol (PEG) nanoemulsions (NEs) [4].

Nucleic acids are large, highly anionic, and enzymatically labile molecules that require cytosolic delivery to be effective [5]. These properties are incompatible with conventional NE design, which was optimized for small, lipophilic molecules [6]. The solution was to construct a lipid system based on an ionizable lipid whose charge changes with pH value [7]. At acidic pH during formulation, the ionizable lipid becomes positively charged and promotes electrostatic complexation with the anionic nucleic acid; at physiological pH, it returns to near-neutral, reducing toxicity and nonspecific protein interactions [7]. The apparent pKa of lipid nanoparticles is a critical parameter for balancing encapsulation efficiency (EE) and endosomal escape [8]. A study by Patel et al. reported that for intramuscularly administered messenger RNA (mRNA) LNPs, the optimal pKa range is 6.6–6.9 [8], while seminal work by Jayaraman et al. established that for small interfering RNA (siRNA)-containing LNPs, optimal hepatic gene silencing occurs within a pKa window of 6.2–6.5 [9].

For conventional NEs stabilized by electrostatic repulsion, an absolute zeta potential of at least 25 mV (|ZP| ≥ 25 mV) is generally considered indicative of adequate electrostatic stability [1,4]. In contrast, ionizable lipid nanoparticles are designed to have minimal surface charge at physiological pH, becoming protonated only at endosomal pH (~5.5) to facilitate endosomal escape [8,9]. The optimal pKa windows for siRNA and mRNA LNPs reflect this pH-dependent charge-switching mechanism [8,9]. Table 1 summarizes the chronological evolution of lipid-based nanocarriers from parenteral nutrition to gene therapy.

### 1.2. The Four-Component LNP Architecture

Modern ionizable LNPs typically contain four main components: an ionizable lipid, a helper phospholipid, cholesterol, and a PEG–lipid. The ionizable lipid enables pH-dependent charge switching, allowing nucleic acid encapsulation at acidic pH while maintaining a near-neutral surface at physiological pH [7,8,13]. The helper phospholipid contributes to bilayer stability. 1,2-dioleoyl-sn-glycero-3-phosphoethanolamine (DOPE), in particular, adopts a cone-shaped geometry that is thought to promote inverted hexagonal phase formation in the acidic environment of the endosome, physically destabilize the endosomal membrane, and facilitate cargo release [13,14]. This is significant in practice: Álvarez-Benedicto et al. showed in a systematic comparison that DOPE-containing LNPs achieve significantly greater endosomal escape than 1,2-distearoyl-sn-glycero-3-phosphocholine (DSPC)-containing LNPs, which are more frequently sequestered in lysosomes after endocytosis [14]. Cholesterol condenses the bilayer and enhances mechanical stability. Cholesterol also influences apolipoptotein E (ApoE) adsorption, which is a major contributor to hepatic uptake in many ionizable LNP formulations. Teng et al. demonstrated this by synthesizing ionizable cholesteryl analogues with reduced ApoE binding, which decreased hepatic accumulation and redirected delivery to the spleen. They also showed that removing cholesterol entirely increased hydrodynamic diameter by 216% [12]. PEG–lipids provide steric stabilization and prolong systemic circulation by applying principles first characterized in PEGylated NE systems, such as reduced protein adsorption and improved physical stability during storage [4,10].

The molar ratio of LNP components is not a secondary formulation detail—it directly determines particle structure and biological function. Cryogenic transmission electron microscopy (cryo-TEM) imaging of several siRNA-loaded ionizable LNP formulations has shown particles with a dense, filled core rather than a hollow shell [15,16]. Because this internal architecture is known to shift with the specific lipids, cargo, and mixing ratios used to build the particle, a filled core should be confirmed for a given formulation rather than assumed to hold across the class as a whole. During assembly at acidic pH, the nucleic acid cargo is sandwiched between closely apposed lipid monolayers, while ionizable lipid not complexed with the cargo coalesces into an amorphous electron-dense core as the pH rises to physiological levels [15]. Interestingly, the helper lipids DSPC and cholesterol behave differently depending on cargo presence: in empty particles, they remain on the outer monolayer, but when siRNA is incorporated during formulation, a fraction of these lipids moves inward with the nucleic acid. This inward movement is necessary for the stable encapsulation of siRNA [16]. The practical implication, often overlooked in formulation work, is that individual lipid components cannot be optimized independently. The internal architecture that determines EE and endosomal escape capacity only emerges from the interactions of all four components during self-assembly [15,16].

## 2. Manufacturing: Microfluidics and the Economics of Scale

### 2.1. Nanoprecipitation and Mixing Fundamentals

LNP formation begins with a deceptively simple idea: rapidly force hydrophobic lipids out of solution so they self-assemble into nanoparticles before aggregating into polydisperse, poorly encapsulating structures. This is accomplished through solvent exchange—a process called flash nanoprecipitation, in which lipid components (ionizable lipids, helper phospholipids, cholesterol, and PEG–lipids) dissolved in ethanol are mixed with an acidic aqueous buffer containing the RNA cargo [17,18,19]. The buffer is maintained at pH 4.0, typically using sodium acetate or citrate, because the acidic environment protonates the ionizable lipids, giving them a transient positive charge. This charge enables electrostatic binding to the negatively charged RNA backbone—a step called flash nanocomplexation, drawing the nucleic acid into the forming particle rather than leaving it free in solution [17].

When the two streams meet, two events occur simultaneously. The sudden change in solvent polarity drives lipid self-assembly, and the electrostatic attraction between the protonated lipids and RNA ensures that the cargo is captured in the process [17,20]. The effectiveness of this process depends almost entirely on the speed and uniformity of mixing. Slow or uneven mixing creates localized regions of high ethanol concentration where lipids aggregate uncontrollably, yielding particles that are excessively large, highly heterogeneous, and poorly loaded with RNA [17,20]. Device geometry further affects mixing performance. In T-junction mixers, the two streams meet at right angles and mixing is driven largely by diffusion. In staggered herringbone mixers (SHMs), asymmetric grooves generate chaotic advection under laminar-flow conditions. In hydrodynamic flow-focusing devices, a central organic stream is confined by flanking aqueous streams, reducing the diffusion distance. These distinct geometries produce LNPs of different sizes, polydispersity index (PDI) and EE even under otherwise identical conditions [21].

To prevent this, mixing must outpace aggregation. Belliveau et al. showed that increasing the total flow rate in SHM devices progressively reduces particle polydispersity, with PDI values falling to 0.03 or below at the highest tested flow rates of 4 mL/min. This demonstrates that faster and more uniform mixing directly results in more homogeneous particles [20]. Leung et al. demonstrated that microfluidic mixing is a general method for encapsulating macromolecules, including mRNA and plasmid DNA, in LNP systems [18]. In practice, these geometries achieve complete mixing quickly enough to consistently produce small, narrowly distributed LNPs. The herringbone groove design of the SHM creates chaotic mixing, accelerating this process compared to simple diffusion [22].

### 2.2. Staggered Herringbone Mixing: Channel Architecture, Particle Control, and Fouling Constraints

The device that made precise LNP synthesis accessible at the lab scale is the SHM, introduced by Stroock et al. [22]. It appears simple—a microchannel with asymmetric herringbone grooves patterned into the floor, but those grooves serve a critical function: they generate chaotic mixing, continuously folding and refolding the two fluid streams around each other under laminar flow conditions, with no turbulence and no moving parts [22]. These characteristics make the SHM particularly well-suited for controlled LNP manufacturing [22,23].

The result is tight, reproducible control over particle properties. Increasing the flow rate ratio (FRR, aqueous to ethanol) or the total flow rate (TFR) predictably reduces particle size, giving researchers a straightforward way to tune formulations [20,23,24]. Belliveau et al. pushed this to its limits, demonstrating that high TFRs progressively reduce PDI to 0.03 or below, yielding increasingly monodisperse particles [20]. Independently, increasing PEG–lipid content from 1 to 5 mol% reduced diameter from approximately 55 nm to about 25 nm [20]. Kimura et al. showed that the microfluidic device for invasive LNP production (iLiNP device) can precisely control LNP size, allowing very fine adjustments within a range of 20 to 100 nm [25].

Microfluidics also shape internal LNP architecture. Cryo-TEM imaging by Leung et al. showed that microfluidically produced LNPs have a nanostructured, electron-dense core consistent with an inverted micellar arrangement, which is distinct from the multilamellar structure of conventional bilayer vesicles [19]. This organization is sensitive to lipid composition: increasing the proportion of the bilayer-forming lipid DSPC, or switching to a more saturated ionizable lipid, shifts the internal structure toward a lamellar organization [19].

The difficulty is that none of this translates easily beyond the lab. The combination of lipids, RNA, ethanol, and water is remarkably effective at fouling microchannel surfaces, particularly the herringbone grooves, and in bare silicon or glass devices, fouling occurs within 10 min, and standard antifouling coating, such as PEG layers, zwitterionic surfaces, or layer-by-layer assemblies, extend this to at most 20 min before the same failure occurs [26]. Standard antifouling coatings provide little benefit; designed for aqueous environments, they deteriorate quickly when exposed to ethanol and aggregating lipids [26]. A more effective solution was recently demonstrated by Hwang et al., who coated the channels of a solvent-resistant silicon/glass chip (SCALAR-AF) with an immobilized layer of perfluorodecalin (PFD), a biocompatible perfluorocarbon with a proven clinical safety record [26]. The resulting slippery, antifouling surface, termed a tethered liquid perfluorocarbon (TLP) coating, repels the LNP mixture rather than allowing it to adhere. The practical impact was substantial: the coated chip operated continuously for over 3 h without fouling or clogging, representing more than a 15-fold improvement over uncoated controls, while producing LNPs with consistent size and EE (>85%) throughout. The chip could also be cleaned and reused [26], making it one of the more convincing demonstrations that microfluidic continuous manufacturing could be viable at scale.

A multi-fold extension of fouling-free channel operation, with EE consistently maintained above 85%, appears to address the underlying problem of LNP component deposition. The unresolved issue is not whether the coating is effective, but whether the process can be scaled. The experiment was conducted on a single chip over only a few hours. GMP-scale production will require either much longer continuous runs or cleaning and reuse procedures that are reliable and reproducible across hundreds of parallel units. These findings show that the TLP coating can substantially reduce component deposition under the tested conditions; however, long-duration performance, cleaning and reuse reproducibility, and GMP-scale scalability remain to be established.

### 2.3. Scalability and Downstream Processing

Scaling up from a lab-scale microfluidic chip to the output required for clinical or commercial supply is a significant challenge. One strategy is to run many channels in parallel. Shepherd et al. integrated 128 SHMs onto a single chip and demonstrated scale-invariant LNP production while maintaining the particle quality typical of single-channel devices [27]. The scale demands of real-world deployment are illustrated by the COVID-19 vaccine rollout: Pfizer and BioNTech delivered 3 billion doses of Comirnaty in 2021 [28], a scale far beyond the capacity of a single microfluidic channel. In principle, this approach works, but it requires nearly perfect flow distribution across all channels; fouling remains an important vulnerability in parallelized systems [26].

For commercial-scale Comirnaty production, Pfizer used a specially designed confined impinging jet mixer, selected for its reproducibility, low fouling, and high throughput [17,28]. Confined impinging jet and multi-inlet vortex mixers are the primary turbulent mixing technologies used for LNP manufacturing at this scale [17]. They operate at Reynolds numbers above 2000—a regime characterized by primarily turbulent flow, with fully developed turbulence above Reynolds 5000—producing mixing at sub-millisecond timescales. This is faster than microfluidic chaotic mixing and avoids the channel clogging issues associated with a micron-scale geometries [17].

When O’Brien Laramy et al. compared the two approaches using an experimental design framework, the turbulent jet mixer outperformed on several metrics: it produced antisense oligonucleotide-LNPs that were consistently smaller, more narrowly distributed, and had higher EE across the tested design space [29]. The particles were not structurally identical; however, microfluidic LNPs had more ordered lipid cores according to small-angle X-ray scattering, while turbulent jet LNPs showed a higher frequency of “bleb” structures according to cryo-TEM [29]. Mixing technology is not just a tool for combining ingredients; it is a critical factor that determines the final product quality.

Regardless of the mixer used, what exits the device is not yet a drug product. The suspension contains approximately 25% (*v*/*v*) ethanol, which is necessary for the process but destabilizes the particles over time and is incompatible with injection [17]. Any residual ethanol must be removed, and the LNPs must be transferred into a physiologically compatible buffer before the product can be used. This adds another unit operation that requires careful process control to avoid damaging the particles [17,30].

### 2.4. Lyophilization

Even a well-manufactured and purified LNP product faces a separate and arguably more significant problem: liquid mRNA-LNP formulations are fundamentally unstable at accessible storage temperatures. The authorized COVID-19 vaccines require storage between −80 °C and −15 °C—a cold chain demand that strains distribution infrastructure, particularly in low- and middle-income countries, and contributes to vaccine wastage [6,31,32]. The underlying issue is water: it drives hydrolysis of the mRNA phosphodiester backbone and promotes lipid degradation, while freeze–thaw cycles during transport can cause particles to aggregate or leak their cargo [30,32,33].

Lyophilization addresses this by removing water and converting the formulation into an amorphous solid in which molecular mobility and, therefore, degradation is greatly reduced [32,33]. The process itself, however, is hostile to LNPs. During freezing, solutes concentrate as ice forms, and the resulting cryo-concentration can drive particle aggregation and fusion. During drying, if conditions are not tightly controlled, water is stripped from the lipid bilayer, causing structural changes that lead to RNA leakage upon reconstitution [30,33].

The standard solution is to add lyoprotectants, typically sucrose or trehalose, before freeze-drying. These disaccharides protect LNPs through two proposed mechanisms: substitution for water by hydrogen-bonding to lipid headgroups (the water replacement theory), or formation of a viscous glassy matrix that physically prevents particles from coming together (the vitrification theory) [30,33]. Ball et al. demonstrated this clearly: LNPs freeze–thawed without lyoprotectants lost substantial activity, while those formulated with 20% (*w*/*v*) trehalose or sucrose survived three cycles with no significant loss of potency and maintained acceptable particle size and PDI. Lower concentrations (5%) provided only partial protection, confirming that the effect is concentration-dependent [30].

The practical ceiling of this approach was demonstrated by Muramatsu et al., who lyophilized nucleoside-modified mRNA-LNPs using a 10% sucrose/10% maltose blend and stored the resulting product at room temperature [32]. After 12 weeks at 25 °C or 24 weeks at 4 °C, luciferase-encoding LNPs maintained functional protein expression in mice comparable to frozen controls, despite a partial reduction in mRNA integrity at 25 °C. An influenza mRNA-LNP vaccine stored under the same conditions induced antibody titers equivalent to the freshly prepared formulation [32]–a result that, if it generalizes, has real implications for how and where mRNA vaccines can be deployed.

The limitation is that lyophilization is slow, batch-dependent, and not easily transferable across formulations. The freeze-drying cycle and lyoprotectant composition must be empirically reoptimized for each new LNP formulation, because changes in lipid composition alter how the system responds to freeze-drying stress [32,33]. The requirement for a separate lyophilization protocol for each new lipid composition is a greater obstacle to clinical translation than the literature typically acknowledges. A lyophilization protocol that can be applied across formulations without reoptimizing the process is not currently available. Tools now in development, such as machine learning (ML), could help address this issue, provided that accurate data on the entire lyophilization process and its outcomes are supplied. Developing a liquid formulation that is stable at room temperature remains an important goal in the field, as it would eliminate the need for lyophilization and its associated process complexities [32,33]. The main manufacturing challenges discussed in this section, along with their underlying causes and proposed solutions, are summarized in Table 2.

## 3. Transdermal LNP Delivery via Dissolving Microneedle Arrays: Fabrication Stress, Stability Gaps, and Limits of Physicochemical Validation

Approved LNP medicines for systemic applications are often administered intravenously, but this method has biological drawbacks that drive the development of alternative approaches [11,34]. Protein corona formation and ApoE-mediated receptor targeting promote rapid hepatic accumulation of LNPs after intravenous injection, with most of the administered dose accumulating in the liver [11,34]. While this hepatic tropism is advantageous for liver-targeted therapies, it severely limits applications requiring extrahepatic delivery [11,34]. In addition to biodistribution concerns, intravenous administration of PEGylated formulations poses a risk of complement activation-related pseudoallergy [35,36]. The skin provides a more accessible option for local and systemic drug and vaccine delivery and has been studied as an alternative route for nucleic acid payloads to bypass the biodistribution constraints of intravenous administration [37,38]. The stratum corneum serves as the main barrier that prevents drug and macromolecules from passively penetrating [37,38]. Microneedles—arrays of the micron-scale projections generally ranging from 150–1500 μm in height [37]—physically breach this barrier without reaching dermal nerve terminals [37,39], creating temporary micron-sized conduits that can enhance the transport of macromolecules and nanoparticles [37,40].

### Critical Limitations of the LNP-Microneedle Platform

The translation of mRNA-LNP microneedle patches to the clinic is hindered by a lack of standardization in mechanical testing protocols, as variations in experimental setup (including whole-array versus single-needle compression testing and differences in Parafilm M-based skin simulant configurations) prevent direct comparison of mechanical performance across studies and complicate the establishment of quality specifications [41]. Harieth Alrimawi et al. further demonstrated that different in vitro test configurations for microneedle mechanical evaluation yield non-comparable results, reinforcing the lack of standardization in this area [42]. Efforts to optimize LNP stability during microneedle manufacturing have shown that polymer choice and drying temperature significantly affect activity, with polyvinyl alcohol outperforming other polymers and 5 °C drying preserving expression better than 25 °C [43]. However, even with these optimizations, LNPs incorporated into dissolving microneedles undergo physical stresses, namely osmotic disruption, mechanical shear during casting, and dehydration, that create a cumulative risk of aggregation, cargo leakage, or mRNA hydrolysis. While functional in vivo delivery has been demonstrated as proof of concept [44], most studies in this field rely on physicochemical metrics such as EE and particle size measured by dynamic light scattering, which indicate structural integrity but do not guarantee endosomal escape or therapeutic efficacy [42,44]. The absence of validated in vivo and regulatory frameworks for LNP-microneedle combination products further exacerbates this translational gap [41].

Osmotic stress, the mechanical force exerted during casting, and water loss during drying are all known to degrade LNP structure [43,44]. There is little reason to believe that the LNP’s ability to escape the endosome after transdermal delivery would withstand these stresses any better than particle size or EE, the two properties actually measured after fabrication. The more reasonable assumption is that manufacturing does compromise endosomal escape capacity to some extent, rather than leaving the particle’s functional properties unchanged while only affecting what is reported. What is missing from the literature is not a reason to doubt that this problem exists, but a direct comparison of escape efficiency before and after incorporation into microneedles, using the same functional assays already established in the endosomal escape literature, instead of relying on particle size and EE as substitutes for whether the particle remains functional [43,44].

The industry considers LNP stability in microneedle matrices an ongoing technological challenge rather than a resolved issue, as indicated by a concurrent patent application filed by Micron Biomedical (Atlanta, GA, USA) and ModernaTX (WO 2025/072510) [45]. Moore et al. conducted ex vivo insertion studies showing that microneedle insertion dynamics differ significantly between mouse and human skin; insertion efficiency in mouse skin was significantly more variable than in human or porcine skin [46]. This variability introduces uncertainty into dose estimates derived from mouse models and should be considered in the design of preclinical LNP-microneedle studies. The current Food and Drug Administration (FDA) guidance on transdermal delivery systems (draft, 2019) does not address nucleic acid payloads [47]. This reflects the fact that no LNP-microneedle combination product has received regulatory approval, though the guidance itself does not state this as a reason. Although proof-of-concept in vivo mRNA expression has been demonstrated in healthy mouse models [43,44], no published study has evaluated LNP-loaded dissolving microneedles in a disease-relevant model, and in vitro–in vivo correlations for this platform remain poorly understood—a gap that defines the field’s most urgent experimental priority. The main barriers to the clinical translation of LNP-loaded dissolvable microneedle platforms are summarized in Table 3.

Beyond patent activity, clinical translation has lagged, with no registered trials identified for LNP-formulated nucleic acid delivered via dissolving microneedles [48,49], despite reported preclinical proof-of-concept for this platform [49,50].

The preclinical landscape for mRNA-LNP delivery via dissolving microneedles has expanded considerably since 2020, with particular emphasis on improving vaccine thermostability and enabling needle-free administration [49,51,52,53]. Hong et al. developed a mannose-modified LNP microneedle system that maintains bioactivity at 4 °C for at least one month and induces pulmonary T-cell responses, providing protection against lung invasion by SARS-CoV-2 pseudovirus in mice [51]. Kim et al. demonstrated a powder attached microneedle platform that preserves mRNA-LNP integrity and maintains stability more than 6 months at 4 °C, significantly reducing off-target liver biodistribution compared with intramuscular injection [52]. Zhao et al. employed a DoE approach to optimize LNP-microneedle fabrication parameters, achieving a transfection efficiency of 43.4% in mesenchymal stem cells and retaining 22.7% activity after 42 days of room-temperature storage [53]. Tang et al. developed a dissolving microneedle patch using a one-step vacuum micromolding process that achieved 95.5% EE and enabled room-temperature storage for at least 30 days, eliciting a comparable IgG response to intramuscular injection at a substantially lower dose [49]. Additionally, a microneedle vaccine printer platform has demonstrated automated fabrication with 6-month room-temperature stability [54]. Collectively, these studies underscore the potential of LNP-microneedle platforms to enhance mRNA vaccine stability and enable needle-free administration. The key characteristics of recently reported representative preclinical mRNA-LNP microneedle platforms are summarized in Table 4.

A search of the ClinicalTrials.gov database [56], conducted on 1 August 2026 using the terms (“lipid nanoparticle” OR “LNP”) AND (“nucleic acid” OR “RNA”), identified 107 clinical studies registered between 2020 and 2026, irrespective of whether the delivery route was local or systemic. Among these, 40 studies were listed as “completed”, 23 as “recruiting”, and 15 as “not yet recruiting”. When the term “microneedle” was added to the search strategy, only one relevant clinical trial was identified (NCT05315362). This Phase 2a proof-of-concept study evaluated intradermal delivery of an LNP-formulated mRNA-1273 vaccine using a solid nanoporous microneedle array. Under the tested loading and delivery conditions, the device was safe but did not induce an anamnestic antibody response, while SARS-CoV-2-specific T-cell responses were lower than after intramuscular administration [57]. The authors attributed this mainly to insufficient vaccine loading and release from the array. This solid-array study should be distinguished from dissolving microneedle systems, for which no registered mRNA-LNP clinical trial was identified. Table 5 summarizes selected ClinicalTrials.gov registered clinical trials involving LNP-based RNA delivery systems, providing insight into the current state of the field, emerging trends, and unresolved challenges from the authors’ perspective.

Table 5 confirms this pattern at scale: across the broader landscape of LNP-based nucleic acid clinical trials, none specifically evaluates dissolving microneedle delivery, and microneedle systems that have advanced furthest in clinical development instead target small-molecule drugs, protein antigens, or inactivated vaccines, with no microneedle-based combination drug product yet approved for any cargo class [43,44,47]. The available evidence therefore establishes proof-of-concept, including room-temperature stability, Th1-polarized responses, and immunogenicity comparable to intramuscular injection, but not clinical readiness. Direct validation of endosomal escape after microneedle fabrication, together with disease-relevant efficiency studies, remains the field’s most urgent experimental priority.

## 4. Design Optimization: From Design of Experiments (DoE) to Machine Learning (ML)

### 4.1. DoE Principles and Application to Lipid-Based Nanoparticle Formulation

Design of Experiments (DoE) is essential in pharmaceutical formulation science, offering a systematic framework for understanding the relationships between formulation variables and product performance. The following example from PEGylated NE development demonstrates the core principles that have since been applied to LNP systems. Using a D-optimal factorial design with variables such as PEG-phospholipid type and concentration, and homogenization pressure and number of cycles, researchers achieved a high coefficient of determination value (R^2^ = 0.9956) for predicting droplet size (Z-ave), indicating that the model accurately captures the effects of these factors and their interactions [4]. This approach produces polynomial models supported by a well-established statistical framework, as demonstrated by ANOVA, R^2^ values, and lack-of-fit testing. By providing coefficient estimates that indicate both the magnitude and direction of each factor’s effect, the model enables formulation scientists to understand not only whether a particular factor is significant, but also why it is important [4].

### 4.2. DoE Limitations for High-Dimensional and Chemically Diverse LNP Formulation Spaces

As formulations become more complex, the limitations of DoE become more apparent. Traditional DoE methods rely on polynomial models that include linear, quadratic, and interaction terms. However, these models can capture non-linearity only if those specific terms are included from the outset. If the relationship between formulation parameters and critical quality attributes (CQAs) is nonlinear, especially with unexpected curvature, experiments that examine only one side of the inflection point can misrepresent the true optimum. Categorical variables pose a particularly challenging problem. The choice of ionizable lipid (D-Lin-MC3-DMA, SM-102, ALC-0315, or a novel candidate), helper lipid (DSPC or DOPE), and buffer composition cannot be effectively represented by a single polynomial model [58]. Each possible combination requires a separate DoE, causing the number of experimental points to increase rapidly as the number of candidate lipids grows. Consequently, if a formulation scientist needs to evaluate several ionizable lipid candidates with different helper lipids, separate factorial designs for each combination quickly become unmanageable [58]. Machine learning (ML) approaches have been proposed as a more flexible alternative capable of handling both continuous and categorical variables simultaneously across large formulation spaces [59].

ML and DoE are better viewed as complementary approaches. ML can help screen large and chemically diverse formulation spaces, while DoE remains useful for local optimization, interaction analysis, process understanding, and experimental confirmation. For instance, Seegobin et al. showed that eXtreme Gradient Boosting (XGBoost) required fewer experimental runs than DoE to identify critical formulation parameters for PLGA nanoparticles [58]. However, several caveats should be considered when extrapolating these findings to LNP systems: the study was conducted on PLGA nanoparticles, which differ fundamentally from LNPs in composition and assembly, and the reported R^2^ of 0.441—while higher than the DoE model’s R^2^ of 0.027—remains modest and does not establish ML superiority for LNP formulation design [58].

More relevant to LNP development, a recent study by Hanafy et al. employed a hybrid workflow that combined DoE-guided high-throughput screening (180 LNP formulations) with ML analysis to identify formulations with selective cellular tropism, with in vivo validation demonstrating redirection of LNP biodistribution from liver to spleen [60]. This LNP-specific study provides more pertinent evidence for the potential of ML in LNP formulation than cross-platform extrapolation from polymeric systems.

Unlike ML models, conventional DoE does not directly learn from heterogeneous datasets reported across the literature, although prior knowledge can still inform factor selection and the boundaries of the experimental design [61]. Therefore, a new DoE optimization effort does not benefit from previously characterized LNP formulations in the literature. DoE remains useful when there are three to five continuous factors and a fixed set of components, but it becomes less effective when there are more than six variables or when the primary goal is categorical lipid selection. These structural constraints define the proper scope of DoE and drive the development of complementary ML techniques [58].

Beyond these general limitations, applying DoE to ionizable lipid design introduces additional complexity related to chemical structure. As Bae et al. showed, ionizable lipids consist of three main parts—a head, a bridge, and tails—and each part can vary in many ways [62]. These variations result in a vast number of possible chemical structures. Traditional DoE is not suitable for exploring such large chemical spaces because it requires selecting a small number of factors to test before starting experiments. In contrast, ML can handle hundreds of chemical features simultaneously to determine which ones are most important for LNP performance. Bae et al. trained a random forest model on 314 features (307 chemical descriptors and 7 formulation parameters) and found that the phenolic group (FP169) was the most important factor for mRNA expression efficiency [62]. For example, FormulationLNP showed that by combining multi-task learning with language model pre-training, it is possible to accurately predict the apparent pKa and in vivo delivery efficiency of LNPs using datasets derived from the literature, thereby mitigating the problem of data scarcity in predicting important LNP parameters [61]. A DoE approach would be impractical for screening this many chemical variations, so researchers would have to test them individually and without any theoretical basis [62]. As noted above, efficiency advantages of ML over DoE have been observed with limited data in certain nanoparticle systems [58].

A further limitation of DoE becomes apparent when the goal shifts from adjusting molar ratios of known lipids to discovering entirely new molecular structures. DoE cannot encode or compare chemical substructures as input variables; it requires quantitative, continuous factors [58,59]. When designing an ionizable lipid from scratch for mRNA therapy, this constraint makes the approach impractical [58,62]. Bae et al. addressed this challenge by training a random forest model on 314 features that encode the chemical substructure of ionizable lipids, along with formulation parameters, demonstrating that ML can map the relationship between molecular structure and LNP performance in a way that DoE cannot [62]. However, DoE remains useful for fine-tuning formulations when the lipid components are already known [58].

This limitation becomes particularly significant when the optimization target is endosomal escape rather than particle size or EE. As established in Section 1.1, the optimal pKa windows reflect the need for pH-dependent charge switching during endosomal acidification [8,9]. Unlike size, which responds predictably to flow rate adjustments, endosomal escape depends on the interplay of these and additional structural parameters (e.g., tail geometry, degree of unsaturation, linker chemistry, and headgroup shape) that interact in ways no polynomial DoE model can adequately capture across a large chemical space [7,8,13,14].

### 4.3. Machine Learning: Algorithms, Data Requirements, and Realistic Benchmarks

Support vector regression (SVR), a kernel-based method, performs well with complex data containing many variables, typically makes accurate predictions on new data, and resists overfitting, making it a suitable option when data is limited. However, it can be difficult to tune and may be computationally demanding [59].

ML algorithms provide a radically new framework for LNP formulation optimization by directly extracting patterns from empirical data, without restricting analysis to predefined models trained on structured data. While this approach increases forecast precision, it may lack interpretability, especially when handling high-dimensional and complex datasets. Therefore, it is important to analyze the advantages and disadvantages of each technique to select the best option for the formulation data in question. The random forest model is robust against overfitting and excels at high-accuracy predictions for highly non-linear problems, making it suitable for datasets with complex variable interactions. However, like most ensemble techniques, it offers less interpretability than linear models [59]. The XGBoost algorithm performs well with large datasets and achieves high forecast precision, but it requires fine-tuning and has low interpretability. ML algorithms such as XGBoost and random forest have proven efficient for predicting mRNA expression efficiency based on ionizable lipid structure and LNP composition parameters [62,63].

Neural networks, such as transformers, can capture complex non-linear relationships but require large amounts of training data and significant computational power [59]. Chan et al. developed a transformer-based neural network called COMET, which integrates molecular structures (encoded via Uni-Mol using atomic types and 3D coordinates), molar percentages, and synthesis parameters (N/P ratio, aqueous/organic mixing ratio) [64]. Trained on the LANCE dataset containing more than 3000 LNPs, COMET achieved a Spearman correlation of 0.873 and a Pearson correlation of 0.866 when predicting transfection efficiency in DC2.4 cells on a random test split. On a more challenging “hits-test” split (withholding the top 10% of formulations), performance remained strong (Spearman 0.725, Pearson 0.820). A key advantage of COMET is its interpretability via integrated gradient analysis. This reveals that lipid identity is the most important predictor—specifically, PEG–lipid choice has the highest influence, followed by ionizable lipid choice (for DC2.4). Molar percentages play a secondary but still significant role, with ionizable and helper lipid percentages being the most impactful among compositional features [64].

FormulationLNP, proposed by Wu et al. [61], addresses limitations of small datasets by employing multi-task learning, in-domain pretraining, and data augmentation. The model includes a chemical language model pre-trained on ionizable lipid molecules using in-domain pretraining and enhanced with SMILES enumeration [61]. FormulationLNP simultaneously predicts two key parameters for LNPs: in vivo delivery efficiency and apparent pKa. On external validation sets, the model achieved an accuracy of 0.829 for in vivo delivery efficiency and 0.656 for apparent pKa, with the lower pKa performance attributed to limited representation of extreme pKa classes in the training data [61].

### 4.4. Performance Benchmarks by Response Variable

Accuracy in predictions from ML models varies significantly depending on the property being predicted and the specific cell line or experiment used. Particle size is among the most easily predictable properties because its underlying determinants are relatively well-understood. Many studies have shown that ML models can predict nanoparticle size with high accuracy [59].

Efficiency predictions for encapsulation vary more widely between research papers. The basic factors behind encapsulation, namely ionic properties of lipids, the amount of nucleic acids, and the charge ratio, are relatively well known; however, different researchers use different assays and methods to measure efficiencies. The accuracy of predictions varies, but ensemble modeling techniques such as random forest and XGBoost can be applied effectively [59].

There are additional challenges related to transfection efficiency and other biological response parameters. According to Chan et al., the ability to predict efficiency depends largely on the cell line used. A formulation that works well for DC2.4 cells may not work at all for B16-F10 cells, and vice versa [64]. In vivo prediction remains particularly challenging due to immune responses, tissue barriers, and weak in vitro–in vivo correlations, which are well documented for LNP systems [64]. Predictions across different species are especially unreliable—a model trained on one cell line or test system usually does not work for another. Based on the articles reviewed above, ML predictions for size and EE appear to be much more reliable than predictions for transfection efficiency when the biological systems differ [59,64]. A model trained on a particular cargo, cell line, formulation platform, or experimental protocol should be treated as a starting point for further optimization rather than as a source of directly transferable design rules.

### 4.5. Batch Effects, Survivorship Bias, and the Interpretability–Accuracy Trade-Off in LNP Machine Learning

#### 4.5.1. Batch Effects and Cross-Laboratory Generalization

The actual size of batch effects in LNP-ML research is currently unknown, as almost no published study has evaluated its model on data from a different laboratory. The Lipid Nanoparticle Database (LNPDB) project illustrates this clearly: Collins et al. showed that the Uniform Manifold Approximation and Projection (UMAP) visualization of LNP embedding data forms clusters based on the lab that produces the data, rather than the actual chemical properties of the particles [65]. In other words, laboratory context predicts outcomes better than formulation chemistry, which is a serious problem for any model trained on aggregated multi-laboratory data. Methodological inconsistencies, such as variations in dose, cell line type, animal model, nucleic acid purity, imaging method, and delivery technique, all contribute to this fragmentation [65]. Collins et al. suggest that including standardized LNP controls, such as Spikevax or Onpattro, in future studies could help mitigate this issue [65]. The genomics field faced a similar issue: batch effects in microarray and RNA-seq data caused widespread irreproducibility for years before the community established harmonization methods and minimum reporting standards. The LNP field has not yet reached such an agreement. Until it does, and until harmonization tools are specifically adapted for formulation data rather than gene expression counts, the safest approach is to subject ML model predictions to rigorous, independent experimental validation before acting on them—a principle explicitly endorsed by both Chou et al. and Collins et al. [63,65].

#### 4.5.2. Survivorship Bias in Training Data

Published research on LNP formulations shows a systemic bias: scientists primarily report successes and rarely publish failures. This results in survivorship bias; i.e., the published record reflects only successful experiments, not those that did not yield favorable results. Collins et al. note that despite considerable effort in assembling LNPDB, the data landscape remains dominated by successful formulations, a direct consequence of publication bias in the field [65]. For ML, this missing data is a significant problem. Training a model only on successful examples introduces bias; it may be overfit to the limited examples it has seen or fail to learn important patterns from what is missing. In either case, the model does not perform well on new data [59]. Researchers identify two main obstacles to building ML models that generalize—fragmented data spread across inconsistent protocols and imbalanced datasets dominated by successful outcomes [65]—both of which undermine model generalizability [59].

Databases such as LNPDB and the LNP Atlas may ultimately be more important for this field than any individual predictive model published so far, because any algorithm trained on fragmented or biased data inherits limitations that even a better architecture cannot overcome. AGILE illustrates this limitation, as detailed in Section 4.6 [66].

#### 4.5.3. Interpretability Versus Accuracy

It remains unclear whether a balance between prediction accuracy and interpretability can be achieved in LNP-ML studies [59,64]. Advanced models such as deep learning provide excellent numerical predictions, but explaining why a particular formulation produces certain results is difficult [59]. This lack of transparency poses problems for regulators and may discourage adoption by clinicians and formulation scientists [59]. Random forest has proven useful for identifying key factors affecting mRNA encapsulation and in vivo expression efficiency through feature importance analysis, e.g., identifying the number of phenol substructures (FP169) as the most important feature for predicting mRNA expression efficiency, with a feature importance score of 0.297 [62]. Transformer models such as COMET achieve outstanding accuracy by focusing on the most relevant parts of the input data, reporting Spearman and Pearson correlations of 0.873 and 0.866, respectively, for predicting LNP efficacy, with t-distributed stochastic neighbor embedding (t-SNE) and integrated gradients used to partially explain model outputs [64]. Selecting the best ML model should therefore depend on the specific task and available data: ensemble methods perform well for highly non-linear problems with abundant data, while hybrid models are preferable when data are scarce or interpretability is required [59].

### 4.6. Hybrid DoE-ML Workflows in LNP Development

Combining traditional DoE with ML provides a practical solution for many areas of the pharmaceutical industry. In this approach, the two technologies work together. ML builds predictive models from data, while DoE designs experiments once new promising candidates are identified. Maharjan et al. developed an LNP-specific hybrid framework using an I-optimal design across 24 mRNA-LNP formulations, XGBoost with Bayesian optimization, and a self-validated ensemble model to predict and optimize critical quality attributes [67]. Bayesian optimization can also be used as a standalone sequential approach for selecting the next experiment and does not necessarily need to be combined with a conventional DoE framework. This approach is particularly advantageous in early-stage formulation development, where drug substance availability and resources are limited. For example, Sato et al. used XGBoost and Bayesian optimization with an initial dataset of 10 data points to identify manufacturing conditions that produced mRNA-LNPs with target particle sizes of approximately 80 or 200 nm and encapsulation efficiencies of 96–97% [68].

The AGILE platform demonstrated how ML can be integrated into a multi-stage experimental workflow, as reported by Xu et al. [66]. Instead of exhaustively screening every compound in a large database, AGILE used a two-step approach. First, it performed a self-supervised pre-training stage on 60,000 computer-generated lipid structures. Then, it finetuned the model using real laboratory experiments on 1200 of those lipids that were actually synthesized [66]. The platform then screened 12,000 candidate lipids in silico using both ML and DoE methods. Completing all three stages in a single day dramatically reduced the time compared to the several months typically required for such experimental work. AGILE outperformed four conventional ML algorithms (Ridge regression, Lasso, Gradient Boosting, and SVM) in both R^2^ and Pearson correlation on the same dataset using the original set of compounds. AGILE also highlights how ML and DoE complement each other. After AGILE identified the best lipids, a separate DoE optimization was conducted for the two most promising candidates. This optimization used a Box–Behnken design with 17 experimental runs [66]. In their review of AI-driven LNP workflows, Su et al. describe a similar logical sequence: ML selects the candidates, and then DoE refines the formulation. This sequence defines an overall artificial intelligence (AI)-driven development approach [69].

Data quality and potential bias in training datasets are a critical limitation for ML-based LNP design, clearly restricting the practical usefulness of these models. Su et al. emphasize that before training ML models on mRNA-LNP data from different experimental batches, researchers must address several issues: inconsistently varying parameters between batches, an excessive number of variables to track, and random measurement errors [69]. In addition, training datasets for mRNA LNP research still contain significant biases in the representation of lipid classes, reducing the ability of ML models developed with these datasets to generalize to new situations. Strategies that may help mitigate the negative effects of small or biased datasets include building physical and chemical descriptors, evaluating screened feature variables, optimizing ML model hyperparameters, and creating bootstrapped resampling methods by generating multiple copies of the training dataset [69]. Similarly, Bhujel et al. reported that 78% of their training dataset consisted of phospholipids with C14-C18 alkyl chains. As a result, phospholipids with unsaturated and branched alkyl chains remain underrepresented, which diminishes the dataset’s generalizability [70]. Xu et al. identified another dataset quality issue with the AGILE platform: the training data came from crude, unpurified lipids, which were produced quickly and in large quantities without thorough purification. Therefore, purifying the lipid materials beforehand is expected to improve the predictive accuracy of future versions of this ML model [66].

The structure–activity relationships that govern endosomal escape, such as pKa, tail geometry, degree of unsaturation, and linker chemistry, are exactly the type of high-dimensional problem identified in Section 4.2. for which ML-based screening is most suitable and classical rational design is least suitable [62,66].

#### Recent Examples of DoE, ML, and Hybrid Workflows in LNP Development

The growing use of computational methods in LNP research is reflected in an increasing number of studies that employ DoE, ML, or their combination to accelerate formulation development and optimize CQAs. Table 6 summarizes recent representative examples, focusing on studies published between 2021 and 2026 that address LNP formulations for nucleic acid delivery.

These examples illustrate a clear trend: traditional DoE remains valuable for screening a limited number of continuous factors, but integrating with ML, or replacing it with ML-driven approaches, enables exploration of much larger chemical and formulation spaces. In particular, ML has been successfully applied to discover novel ionizable lipid from virtual libraries, predict biological performance from structural features, and optimize manufacturing parameters with fewer experimental runs. Bayesian optimization, used either in hybrid workflows or as a standalone sequential design tool, offers an efficient strategy for navigating high-dimensional parameter spaces by iteratively selecting the most informative next experiment. Collectively, these advances point toward a future in which computational screening and experimental validation are tightly integrated, reducing development time and cost while improving the likelihood of identifying clinically translatable LNP formulations.

## 5. Biological Barriers to LNP Efficacy

### 5.1. The Endosomal Escape Problem in Nucleic Acid Delivery

LNPs have emerged as one of the most effective platforms for delivering nucleic acid therapeutics, as demonstrated by the COVID-19 mRNA vaccines and patisiran, an siRNA-based drug [77]. However, despite these successes, LNPs face a fundamental challenge that limits their full potential: releasing the nucleic acid cargo from endosomes into the cytosol, where it can exert its effect [77,78,79]. When LNPs enter a cell, they are sequestered inside membrane-bound compartments called endosomes, which serve as cellular “holding chambers”. If the nucleic acid does not escape from these compartments in time, it is degraded in lysosomes or expelled from the cell, never reaching its target. This process, known as endosomal escape, is widely regarded as the primary bottleneck in nucleic acid therapy [78,79,80,81,82].

The 1–2% escape efficiency originally measured by Gilleron et al. using electron microscopy of gold-labeled siRNA has since been examined from multiple independent perspectives, and none offer a more optimistic outlook [78]. Liu et al. developed a specialized reporter system based on the SNAPswitch probe that directly measures cytosolic mRNA delivery without being confounded by the total amount of mRNA entering the cell or the amount of protein eventually produced [80]. Using this method, they found that for every commercial LNP they tested, including SM-102 (Spikevax), DLin-MC3-DMA (Onpattro), and ALC-0315 (Comirnaty), less than 10% of the mRNA successfully escaped from the endosome into the cytosol [80]. More surprisingly, the level of protein expression did not correlate well with the amount of mRNA that actually entered cells or with the amount delivered to the cytosol; SM-102 elicited substantially higher protein expression per unit of cargo delivered to the cytosol compared to DLin-MC3-DMA, indicating that factors downstream of endosomal escape, such as mRNA stability, access to ribosomes, and translation kinetics, introduce additional limitations that most LNP optimization efforts have not systematically addressed [80].

Mrksich et al. traced the evolution of lipid design from permanently charged cationic lipids to ionizable structures with pKa values in the 6.2–6.5 range, showing how this shift enabled more efficient membrane disruption via the hexagonal HII phase transition [83]. However, the review also makes clear that pKa alone is not a reliable predictor of escape; lipid tail geometry, degree of unsaturation, linker structure, and headgroup chemistry all independently affect how well a lipid can fuse with and destabilize the endosomal membrane as the endosome becomes more acidic [83]. No single structural parameter predicts escape across different formulations. This complexity is precisely why the structure–activity space for ionizable lipids is a natural target for ML-based screening: it is simply too intricate for classical rational design to navigate efficiently [83]. Separate live-cell imaging experiments by Wittrup et al. confirmed this, finding that only about 3.5% of LNP-containing vesicles successfully release their cargo, and that effective gene silencing was achieved with as few as approximately 2000 cytosolic siRNA copies per cell [79]. Even ionizable lipids already approved for clinical use perform poorly by this measure. Liu et al. found that SM-102 showed approximately 20% higher cytosolic delivery than DLin-MC3-DMA and eightfold higher than ALC-0315. When normalized to cellular association, however, the estimated endosomal escape efficiencies remained low, at approximately 10%, 5%, and 4%, respectively [80]. Three independent measurements have been conducted: image-based cell imaging, live-cell fluorescence tracking using Rab5/Rab7 markers, and a cytosolic reporter assay. These studies use different experimental endpoints and are not directly interchangeable. Taken together, they indicate low functional release in the systems examined, but they do not establish a universal ceiling for all ionizable LNPs. None of these studies determines whether the low escape efficiencies observed are specific to the tested lipids (DLin-MC3-DMA, SM-102, ALC-0315) or to the escape mechanism itself, regardless of the lipid used. If the ceiling is lipid-specific, a better-designed ionizable lipid should eventually surpass it. If the ceiling instead reflects something inherent to vesicle collapse and membrane fusion, further progress in lipid design alone will not suffice, and the field will need to look beyond structural optimization of the lipid itself. This decoupling suggests that what happens after the cargo reaches the cytosol matters at least as much as the escape event itself [80]. These findings support the conclusion that endosomal escape is a critical barrier to efficient nucleic acid delivery [77,78,79]. However, the reported efficiencies are expressed relative to the amount of cargo internalized by cells, not relative to the total quantity of LNPs administered systemically. The relationship between cellular uptake, tissue distribution and endosomal escape is complex, and escape measurements cannot be used to estimate what fraction of the injected dose ultimately reaches the cytosol. Consequently, the translational implications of this bottleneck—including the doses required for therapeutic effect and the potential for off-target activity—can be assessed only by integrating pharmacokinetic, biodistribution, and cellular uptake data, not by escape efficiency alone.

**Figure 1:**
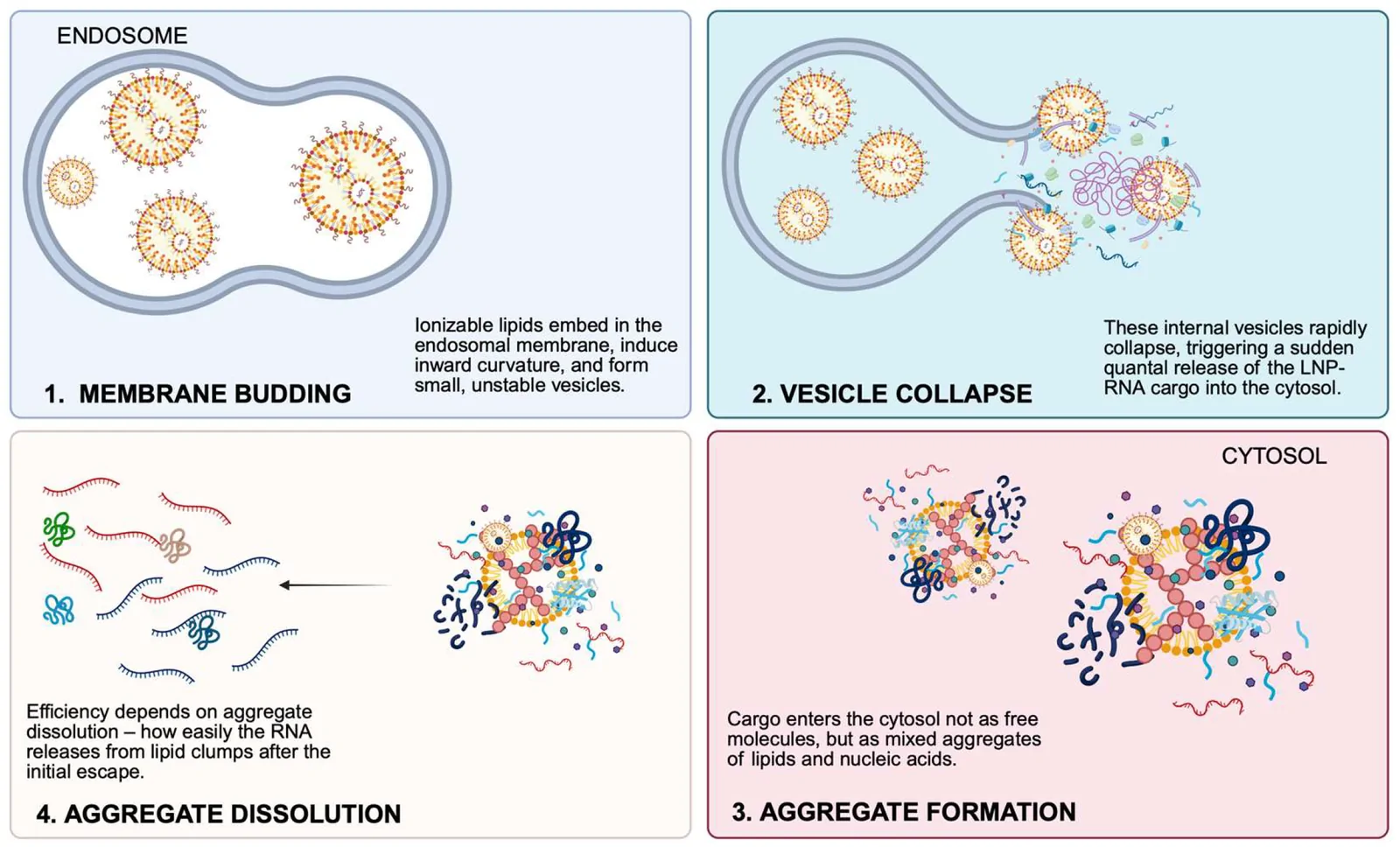
Proposed Vesicle Budding and Collapse (VBC) model of endosomal escape. (**1**) Membrane budding: ionizable lipids embed in the endosomal membrane and induce inward curvature. (**2**) Vesicle collapse triggers quantal release of cargo. (**3**) Aggregate formation: cargo enters the cytosol as mixed lipid–nucleic acid aggregates rather than as free molecules. (**4**) Aggregate dissolution is proposed to be a rate-limiting step in functional delivery. Created with BioRender.com.

The release of LNPs from endosomes has been studied for many years, and it is now clear that this is a complex, multistage process that remains incompletely understood [77,84]. Initially, two simple hypotheses were proposed: either the LNP binds protons, leading to endosomal swelling and destruction (“proton sponge” effect), or the LNP fuses directly with the membrane. However, further studies showed that neither model is fully validated nor sufficient to explain all experimental observations. The actual process is more complex [84]. A key factor is the pKa of ionizable lipids (6.2–6.5 for siRNA and 6.6–6.9 for mRNA, as established in Section 1.1), which drives a dramatic conformational change inside the acidic endosome, from a flat, layered structure to a cone-shaped inverted hexagonal form. This new shape facilitates fusion of the LNP with the endosomal membrane and release of the nucleic acid cargo. As discussed in Section 1.2, the choice of helper lipid directly determines how effectively ionizable lipid geometry translates into membrane fusion: DOPE promotes the negative curvature that drives bilayer destabilization, whereas 1,2-dioleoyl-sn-glycero-3-phosphocholine (DOPC) stabilizes flat lamellar structures and is substantially less fusiogenic [14,80]. A more recent mechanistic framework, the Vesicle Budding and Collapse (VBC) model, was proposed by Pei [84] to explain the sequence of events leading to endosomal escape. It should be emphasized that this framework currently remains a working hypothesis: the principal publication describing the model is a perspective article, and the individual steps of the proposed pathway—particularly budding of the endosomal membrane and the subsequent collapse of the forming vesicle—have not yet been visualized directly in living cells. With these caveats in mind, the model proposes that the destabilized LNP releases free ionizable lipids that embed in the inner leaflet of the endosomal membrane. Because these lipids have an inverse cone shape, they cluster, induce inward membrane curvature, and form a small, unstable vesicle that then rapidly collapses and releases a mixed lipid–nucleic acid aggregate into the cytosol [84]. This four-stage sequence is shown in Figure 1. This model may account for several observations from live-cell studies, including the sudden quantal release of cargo (rather than gradual leakage), the rapid recruitment of galectin proteins to injured endosomal membranes, and the subsequent aggregation of lipid and nucleic acid into a perinuclear non-vesicular structure [79,84].

Several studies have examined the narrow “window of opportunity” during which macromolecules such as siRNA can escape from endosomes into the cytosol, reaching similar conclusions about how brief this period is. For example, Wittrup et al. [79] used fluorescent markers to track endosomes and determined that siRNA can escape from lipoplexes and LNPs only within 5–15 min after the particles enter the cell. They found that this window opens after endosomes lose the early marker EEA1 but before they acquire the late marker LAMP1. During this interval, two small proteins, Rab5 and Rab7, are present on the endosomes. These findings are consistent with those of Gilleron et al. [78], who observed that LNPs accumulate in a hybrid endosomal compartment exhibiting both early and late endosomal characteristics before cargo escape occurs. Once an endosome becomes LAMP1-positive and starts to mature into a lysosome, the window of opportunity closes permanently. Any cargo still trapped inside will be degraded by the lysosome [78,79].

All of this indicates that the escape of macromolecules or siRNA from endosomes is not a random process but a carefully regulated mechanism that depends on the precise biochemical state of the maturing endosome. Another important insight is that endosomal escape is necessary but not sufficient for successful delivery. A second limitation occurs after vesicle collapse, involving a process called aggregate dissolution, in which the cargo is released from insoluble clumps that form upon vesicle collapse [80,84]. This helps explain why some ionizable lipids have similar rates of endosomal escape but very different functional outcomes, as shown by Liu et al. [80]. In their study, SM-102 and DLin-MC3-DMA had comparable endosomal escape rates, yet SM-102 produced more than ten times higher levels of protein expression than DLin-MC3-DMA [80]. The superior performance of SM-102 is likely due to its structure; SM-102 has branched alkyl chains, which form less tightly packed clumps after vesicle breakdown, allowing the mRNA cargo to be released more rapidly and completely [80,84]. Similarly, replacing cholesterol with beta-sitosterol improved efficacy through two mechanisms: increased cellular uptake and more efficient cargo release from post-escape aggregates, but not through enhanced endosomal escape, which was virtually identical between the two sterols [80,84]. The bulkier, more branched structure of beta-sitosterol leads to looser lipid packing, which facilitates cargo release [80]. Therefore, functional delivery efficiency results from two sequential processes: endosomal escape and subsequent aggregate dissolution [80,84].

Another aspect of the endosomal escape problem that has received less attention than efficiency is the impact of the escape event on the cell. Omo-Lamai et al. showed that the same membrane disruption that enables mRNA to exit the endosome also triggers inflammation, and that these two outcomes are difficult to separate with conventional ionizable lipids [85]. The key variable is the size of the membrane perforation. Perforations small enough to be repaired by the endosomal sorting complexes required for transport (ESCRT) machinery resolve without significant inflammation while still allowing cargo release. Larger perforations that exceed ESCRT repair capacity expose luminal glycans, which recruit galectins and initiate downstream inflammatory signaling. Specialized ESCRT-recruiting lipids or galectin inhibitors can exploit this distinction to decouple efficient cargo delivery from inflammatory signaling [85]. This reframes the design challenge: the goal is not to maximize membrane disruption, but to calibrate it, creating perforations large enough to release mRNA yet small enough to be repaired. This introduces a structural design constraint that pKa optimization alone cannot satisfy [85].

Overcoming these barriers will require more than incremental lipid modifications. Established design principles—matching pKa to the endosomal pH range of 6.2–6.8, incorporating branched hydrophobic tails, selecting DOPE as a helper lipid to favor inverted hexagonal phase formation, and titrating PEG–lipid content to balance colloidal stability against fusogenicity—provide a starting point [77,80,84]. What remains unresolved is the mechanistic detail: whether ESCRT proteins or SNAREs participate in VBC has not been established, and direct real-time imaging of the VBC process in living cells has not yet been achieved [84]. Standardized assays capable of measuring both escape yield and aggregate dissolution efficiency across formulations would allow these questions to be addressed systematically and would provide the translational bridge needed for therapies targeting organs beyond the liver [84].

### 5.2. Protein Corona and the Revision of the ApoE Model

When LNPs enter the bloodstream, plasma proteins begin adsorbing to their surface almost immediately. The resulting protein corona is not a uniform coat; it has structure. Proteins that bind tightly and irreversibly form the “hard corona”, which develops within seconds and largely determines the particle’s destination in the body. Opsonins such as immunoglobulin G and complement proteins are prominent components and promote uptake by the mononuclear phagocyte system. A second, loosely bound layer, the “soft corona”, exchanges continuously with the surrounding fluid and influences how cells initially recognize the particle [86]. Walkey et al. showed that when nanomaterials enter a physiological environment, they rapidly adsorb proteins from the surrounding fluid, forming a protein corona that fundamentally alters their biological behavior and determines how they interact with cells and tissues [87]. Lundqvist et al. showed that the identity of the proteins recruited into the hard corona is governed primarily by particle size and surface chemistry, findings that have direct implications for how biological systems subsequently recognize and interact with the particle [88]. Monopoli et al. [89] took this further, arguing that the biological environment actually responds to the hard corona, not to the nanoparticle surface beneath it. They called this the nanoparticle’s “biological identity”, meaning that cells and tissues interact not with the nanoparticle itself, but with the protein layer that forms around it [89].

For LNPs specifically, comparisons of hepatic uptake and gene silencing in wild-type versus ApoE-knockout mice identified ApoE adsorption as the molecular event underlying the liver tropism of ionizable LNPs [90]. Francia et al. subsequently reviewed this and related evidence, noting that corona composition is also influenced by formulation variables such as PEG–lipid density and surface charge [91]. This also reflects a broader limitation in the field: whether attaching targeting ligands to LNPs meaningfully improves clinical outcomes over simpler formulations remains debated, since few studies have systematically compared particles with and without a ligand. Part of the difficulty is that the protein corona can mask targeting ligands, preventing them from binding their intended receptor even when the ligand itself is intact and exposed [92]. For this reason, including a ligand does not automatically increase LNP targeting, and dedicated comparisons of ligand-bearing and ligand-free formulations are therefore needed to establish whether the ligand provides a genuine targeting benefit. PEG–lipid surface density and chain length are key formulation parameters that shape LNP corona composition: increasing PEG content has been shown to reduce protein binding to the LNP surface [93], whereas longer, more persistent PEG chains such as PEG-C18 can, conversely, reduce hepatocyte uptake and gene silencing compared with shorter, detachable PEG-C14 chains [90]. The optimal PEG–lipid content therefore represents a balance between these competing effects. The explanation that LNPs accumulate in the liver because they bind to ApoE, which then attaches to LDL receptors on hepatocytes, is only partially accurate. Dilliard et al. conducted organ-selective targeting studies and found that altering the lipid composition of LNPs changes which serum proteins are recruited to their surface, thereby redirecting organ distribution. Liver-targeting LNPs associate with ApoE, while lung- and spleen-targeting formulations associate with other proteins such as vitronectin and beta2-glycoprotein I, suggesting that mechanisms beyond ApoE contribute to organ-specific delivery [11]. Nevertheless, ApoE remains essential for hepatic uptake. Van Straten et al. showed that heat inactivation of serum degrades ApoE, which significantly reduces the uptake and cargo delivery of DLin-MC3-DMA LNPs in vitro, confirming its central role in mediating LNP internalization by liver cells [94]. Feng et al. reviewed evidence suggesting that disease-associated changes in plasma proteins may alter nanoparticle corona composition and biodistribution. However, the extent to which these effects apply across different LNP formulations remains to be established [95].

**Figure 2:**
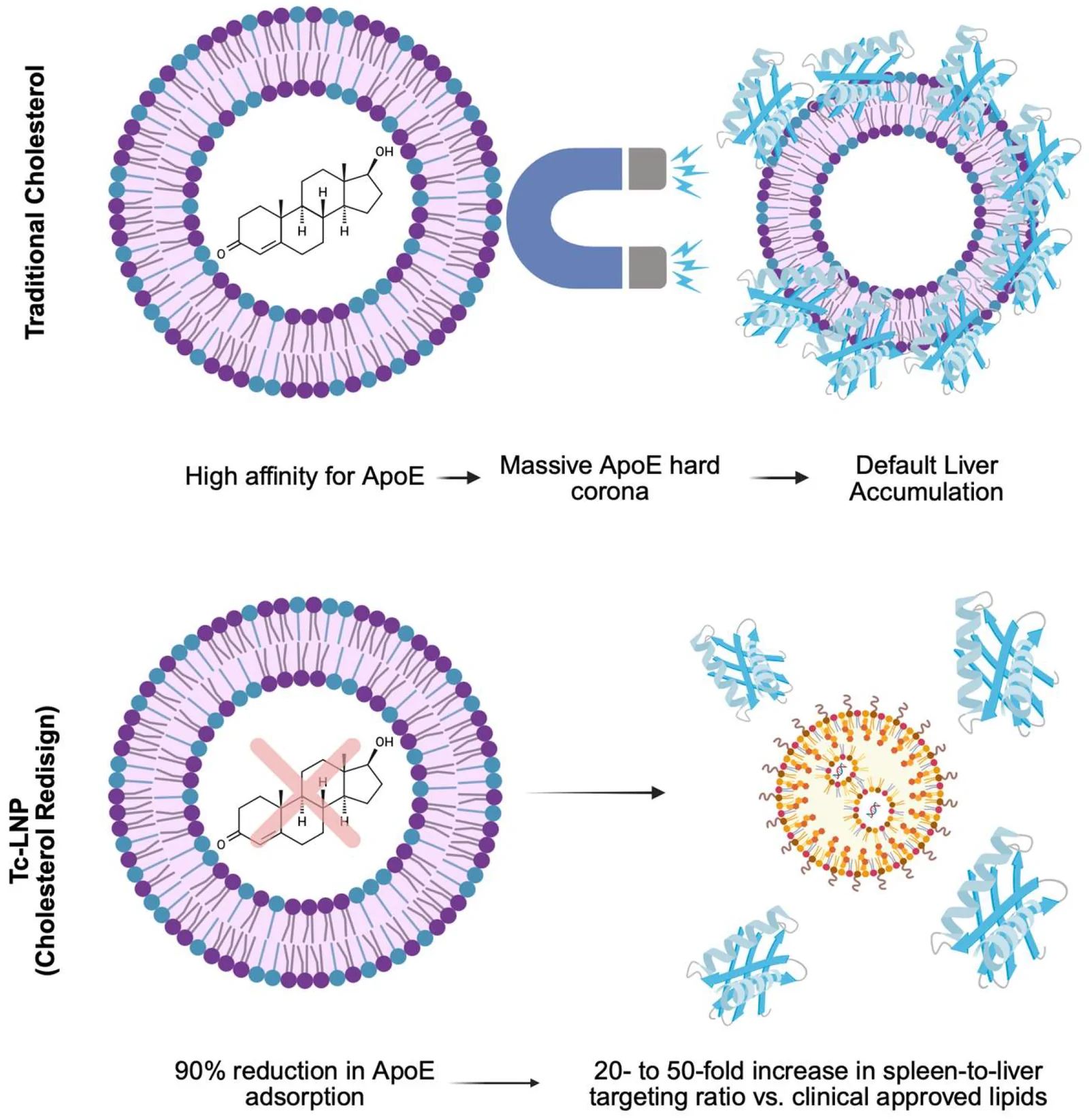
Cholesterol redesign strategy underlying the Tc-LNP platform. (**top**) Conventional cholesterol has a high binding affinity for apolipoprotein E (ApoE), generating a dense, ApoE-rich hard corona that drives default hepatic accumulation via LDL receptor uptake. (**bottom**) Replacing cholesterol with a redesigned analogue (Tc-LNP) reduces ApoE adsorption by approximately 90%, resulting in a 20- to 50-fold increase in the spleen-to-liver targeting ratio compared to clinically approved lipids. Created with BioRender.com.

The clearest evidence of how ApoE and LDL receptors work together to target hepatocytes comes from Teng et al. [12], whose three-component LNP (Tc-LNP) strategy was introduced in Section 1.1. These Tc-LNPs adsorbed approximately 90% less ApoE than traditional LNPs, resulting in significantly reduced hepatic accumulation and increased extrahepatic delivery, especially to the spleen. The spleen-to-liver luminescence intensity ratios for the top two Tc-LNPs were 4.78 and 5.20, representing a 20- to 50-fold increase compared to the clinical lipids DLin-MC3-DMA and ALC-0315, which achieved ratios of only 0.24 and 0.12, respectively. This confirms successful redirection from the liver to extrahepatic organs through rational cholesterol redesign [12]. This work changes our understanding of cholesterol: it is not just a passive structural building block, but also actively determines where LNPs go in the body. Figure 2 illustrates this cholesterol redesign strategy and its effects on ApoE adsorption and organ distribution.

Teng et al. [12] based their Tc-LNP work on a specific, pre-stated hypothesis: reducing the lipid’s affinity for ApoE should reduce hepatic accumulation. They tested this prediction directly by synthesizing cholesterol analogues with deliberately altered ApoE binding, and the results matched the hypothesis. Selective organ targeting (SORT) is more difficult to evaluate in the same way. The proposed mechanism to explain LNP selectivity for organs other than the liver is altered serum protein binding, depending on which supplemental lipid is used. However, it is not clear from the published literature whether this protein-binding mechanism was anticipated before the relevant lipids were tested or was worked out afterward to explain an assumption already made. This distinction is significant for anyone trying to apply either approach to a new organ. A mechanism predicted in advance can guide the design of the next lipid, while a mechanism reconstructed afterward will have to be rediscovered, organ by organ, through another round of empirical screening. Therefore, systematically studying cholesterol analogues may offer a practical way to develop LNPs that target organs beyond the liver, while avoiding the complexity and immune reactions associated with surface-attached targeting molecules.

## 6. Future Directions

### 6.1. Synthesis of Cross-Cutting Limitations in Manufacturing, Delivery, Computation, and Biology

This review has examined six interconnected areas of LNP development—microfluidic manufacturing, lyophilization, transdermal microneedle delivery, ML-guided formulation, endosomal escape, and protein corona-mediated targeting—through the common lens of the gap between routinely measured endpoints and functional therapeutic performance. In manufacturing, channel fouling during microfluidic production and process control during tangential flow filtration are documented operational bottlenecks, yet neither is systematically reported alongside the particle quality attributes they affect [21,26,96,97]. Lyophilization protocols remain formulation-specific and cannot be transferred between ionizable lipids without empirical re-optimization, a constraint that is underappreciated given the scale of cold-chain dependence in current mRNA vaccine distribution [30,31,32,33]. In microneedle-LNP systems, fabrication stresses are known to compromise particle integrity, but whether they also impair endosomal escape function—the step that determines therapeutic activity—has never been directly tested [42,43,44]. In ML, the generalizability problem stems not from algorithmic limitations but from the input data: survivorship bias and laboratory-specific batch effects constrain model performance in ways that no computational advance can overcome without better-structured training sets, as discussed in Section 4.5 [63,65]. At the biological level, the ApoE/LDL receptor model of hepatic accumulation is now known to be incomplete—organ-selective delivery depends on a broader ensemble of serum protein interactions, as detailed in Section 5.2 [11,91,92]. In cited cellular studies, escape estimates remained approximately 1–2% for selected siRNA-LNP systems and below 10% for the tested mRNA-LNP formulations [78,79,80]. Finally, rational redesign of the cholesterol component alone, without surface-attached targeting ligands, has been shown to redirect biodistribution from liver to spleen by 20- to 50-fold, supporting lipid engineering as a promising and potentially scalable route to extrahepatic delivery [12].

These barriers share a common feature: they become visible only when functional biological validation is required alongside physicochemical characterization. The seven priorities proposed in Section 6.2 are the minimum methodological corrections needed to close the gap. Some of these corrections are closer to implementation than others. Table 7 distinguishes between them.

### 6.2. Future Research Priorities

**Figure 3:**
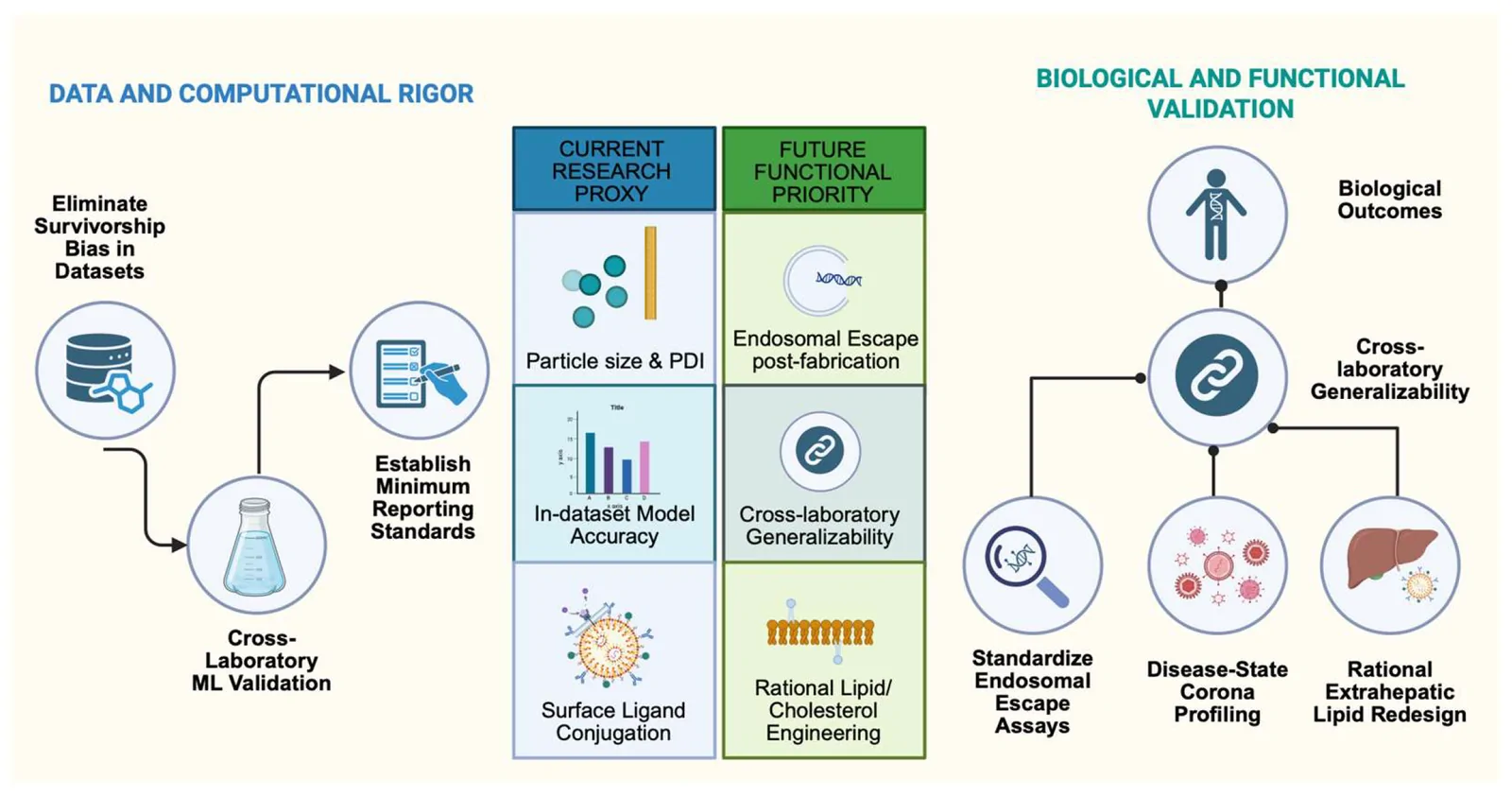
The future research priorities organized into two complementary domains. (**left**) Data and computational rigor: eliminating survivorship bias in training datasets and establishing minimum reporting standards are prerequisites for meaningful cross-laboratory machine learning (ML) validation. For each current physicochemical proxy—particle size and polydispersity index (PDI), in-dataset model accuracy, and surface ligand conjugation—a corresponding functional priority is identified: endosomal escape post-fabrication, cross-laboratory generalizability, and rational lipid/cholesterol engineering, respectively. (**right**) Biological and functional validation: standardized endosomal escape assays, disease-state corona profiling, and rational extrahepatic lipid redesign converge on cross-laboratory generalizability as the link between mechanistic studies and reproducible biological outcomes. Created with BioRender.com.

Figure 3 groups the future research priorities proposed in this review into two complementary domains: data and computational rigor, and biological and functional validation.

#### 6.2.1. Resolve the Manufacturing Bottlenecks That Limit Reproducibility Across Scales

Channel fouling, buffer sensitivity, downstream ethanol removal, and tangential flow filtration process control are documented operational problems that receive insufficient formal attention in the literature [26,96,97]. The field needs agreed-upon in-process checkpoints: TFR and FRR values should be reported with particle size and PDI as mandatory parameters rather than optional supplementary data, because without them, manufacturing conditions cannot be reproduced across laboratories [21,96]. Parallelization strategies for scale-up, whether through multi-channel microfluidic arrays or impinging jet mixer systems, require standardized characterization protocols to confirm that CQAs are maintained as production volume increases, as demonstrated for COVID-19 vaccine manufacturing [17,27,28]. For lyophilized formulations, cryoprotectant type, concentration, buffer composition, and ionizable lipid identity must be re-optimized for each new formulation, and functional potency after reconstitution must be reported with physicochemical parameters [30,31,32,98].

#### 6.2.2. Functional Validation of LNP Integrity Following Microneedle Fabrication

Microneedle-based delivery is included in this review not as a tangential topic, but because it integrates the manufacturing, formulation, and biological challenges discussed above into a single unresolved problem: LNPs must survive the fabrication process, retain their endosomal escape function, and be delivered through an entirely different route, all simultaneously. As detailed in Section 3, the cumulative physical stresses of microneedle manufacture create risks that routine physicochemical testing is not designed to detect, and the field has not yet advanced beyond proof-of-concept studies in healthy mouse models [43,44]. Two specific methodological gaps follow directly from this. First, the lack of harmonized mechanical testing protocols means that results cannot be meaningfully compared across studies, and quality specifications for loaded microneedles cannot be established on a reliable empirical basis [43]. Second, and more fundamentally, demonstrating physicochemical integrity after fabrication is not equivalent to demonstrating functional activity—whether fabrication stress silently compromises endosomal escape capacity in ways that particle size and EE measurements cannot detect remains an open question that no published study has directly addressed [42,43,44,78,79]. Resolving this will require functional assays and disease-relevant in vivo models, not buffer dissolution experiments alone [43,44,46].

#### 6.2.3. Cross-Laboratory Machine Learning Validation as a Primary Research Objective

As shown by Collins et al. [65], batch effects in existing LNP datasets are not hypothetical. The authors found that LNP formulations cluster by study origin rather than by chemical composition, and that within-study comparisons are significantly more similar than across-study comparisons, indicating that experimental variability between laboratories can overshadow structure–function relationships [65]. A study that tests the same ML model on independently collected data from multiple laboratories using identical protocols, while systematically tracking potential sources of batch variation, would provide the field with the performance benchmarks it currently lacks [65].

#### 6.2.4. Direct Quantification of Endosomal Escape Efficiency

As discussed in Section 5.1, there is no standardized assay for measuring endosomal escape efficiency, and the two most-cited estimates, obtained by fundamentally different methods, are not directly comparable [78,79]. Gilleron et al. used electron microscopy to visualize gold-labeled siRNA and estimated that only 1–2% of internalized siRNA escapes from endosomes into the cytosol [78], while Wittrup et al., using high-dynamic-range fluorescence imaging, estimated that approximately 2000 cytosolic siRNAs per cell are sufficient for maximal knockdown [79]. This disagreement is not merely technical; it reflects the absence of a standardized assay that can be applied across laboratories, formulations, and cell types under realistic conditions [80,81]. The problem is further complicated by the fact that commonly used proxies for endosomal escape, namely cellular uptake and protein expression, do not reliably indicate escape efficiency. Liu et al. showed that uptake levels did not correlate with mRNA expression across a panel of LNP formulations, and that all tested formulations achieved less than 10% escape efficiency despite substantial differences in their physicochemical properties [80]. Chatterjee et al. confirmed that endosomal escape remains a fundamental bottleneck, not a solved problem, and that the field lacks mechanistic tools to distinguish between formulations that genuinely improve escape and those that merely increase uptake or expression through other mechanisms [81]. The field therefore needs a direct, single-molecule-sensitive detection method that does not require labeling the cargo in ways that could alter its intracellular behavior. Until such a method is established and widely adopted, claims that a given lipid or formulation “improves endosomal escape” should be treated with caution, as they typically rely on indirect proxies such as protein expression or gene silencing rather than escape efficiency itself [80,81].

#### 6.2.5. Protein Corona Characterization Under Disease-State Conditions

The protein corona that forms on LNPs is not fixed; its composition varies with the individual’s physiological and pathological state, because the plasma lipoprotein profile determines which proteins are available to form the corona [86,95]. Comparisons of hepatic uptake in wild-type and ApoE-knockout mice have shown that ApoE adsorption onto the LNP surface is essential for its liver tropism [90]. Disease conditions can profoundly alter the composition of plasma proteins available for corona formation, and the corona formed in patient plasma may therefore differ substantially from that formed in plasma from healthy volunteers [86]. Nevertheless, most preclinical LNP studies characterize corona composition using plasma from healthy individuals, even though target patient populations often have conditions such as obesity, hypercholesterolemia, hepatic steatosis, or cancer [86,95]. To account for patient-to-patient variability, researchers should systematically characterize the corona using plasma from relevant patient populations before clinical trial design, by quantitatively profiling corona-associated proteins and assessing LNP uptake in disease-relevant cells [86].

#### 6.2.6. Rational Extrahepatic Targeting Through Lipid Engineering

As discussed in Section 5.2, the conventional approach of attaching targeting ligands to the LNP surface after synthesis has fundamental limitations: the protein corona can bury ligands before the particle reaches its intended tissue, and the attached molecules alter corona composition in unpredictable ways [91]. As shown by the SORT and Tc-LNP studies discussed above, organ selectivity can be programmed through formulation chemistry alone, without post-synthesis conjugation [11,12,99]. Systematically extending these principles across a broader chemical space of cholesterol analogues, ionizable lipid tail geometries, and supplemental charged lipids offers a more scalable and immunologically straightforward path to extrahepatic delivery than surface functionalization [12,99].

#### 6.2.7. Systematic Collection of Negative Formulation Results

As detailed in Section 4.5, both LNPDB and the LNP Atlas rely exclusively on published peer-reviewed literature, which is biased toward successful formulations [65,100]. Models trained on this data inherit the same bias rather than correcting it. Prospectively depositing formulation data (before study beginning and regardless of outcome, similar to clinical trial registration) remains the only way to address this problem at its source. No such standard has been established in LNP research [63,65].

### 6.3. Open-Access LNP Formulation Database

Database infrastructure is considered a separate research priority here because it is a prerequisite for all ML-based approaches discussed in Section 4, rather than an extension of any single algorithm. Without harmonized, bias-corrected training data, computational advances in model architecture cannot meaningfully improve real-world generalizability. Researchers in the LNP field generate formulation data much faster than the community can organize or use it. To address this problem, Collins et al. created LNPDB, a large collection containing 19,528 well-standardized records [65]. This project demonstrated both the potential of a central LNP database, and the significant challenges involved, especially inconsistencies in experimental methods. Comparing results is difficult when studies use different doses, cell types, animal models, nucleic acid purity levels, or imaging technologies [65]. Song et al. independently created the LNP Atlas, a repository with 1092 LNP formulations collected from 63 peer-reviewed papers published between 2005 and 2025 [100]. The Atlas is highly detailed, recording lipid types (with SMILES codes), molar ratios, physicochemical properties (particle size, PDI, ZP, EE), synthesis details, and bioactivity profiles [100]. Both LNPDB and the Atlas use only data from published studies, which mostly report successful outcomes [65,100].

Given these challenges, a staged approach is recommended. The most urgent step is for the field to agree on minimum reporting standards, ideally enforced by journals through a formal checklist at submission. At a minimum, this should include the type and amount of ionizable lipid, helper lipid, and cholesterol; the type and amount of PEG–lipid; the manufacturing method and its key parameters, particle size, PDI, and ZP; EE together with the specific method used to measure it; and the type and sequence of the nucleic acid cargo [65,100]. Without this baseline consistency, even the best-curated database will continue to inherit the fragmentation already present in the published literature [65,100].

Building on this foundation, resources like LNPDB and the Atlas can grow only if researchers voluntarily extract and contribute their own published data [65,100]. This process becomes much more manageable if groups adopt shared templates, such as the one introduced by Song et al., rather than formatting data independently [100]. Once sufficient data accumulates in this standardized form, it becomes feasible to build a web-based prediction tool capable of estimating particle size and EE directly from formulation parameters [65]. Such a tool could also provide confidence estimates for its predictions to help users assess reliability. An accompanying application programming interface (API) would allow this predictive capability to be queried programmatically, enabling researchers to integrate it directly into their own ML pipelines [65].

Chou et al. highlight a persistent challenge: models trained on cell culture data often fail to translate to living organisms. This discrepancy occurs because in vivo systems introduce additional complexity not present in vitro, such as immune responses, physical tissue barriers, and off-target effects [63]. Both databases acknowledge that performance metrics may vary significantly between studies because of differences in experimental conditions, characterization methods, and biological assay systems. Users are cautioned not to treat compiled data as directly comparable across sources [65,100]. Song et al. [100] caution that users should always consult the original publications for experimental details, as results can vary significantly depending on how an experiment was conducted, how outcomes were measured, and which biological system was used [100].

Both LNPDB and the LNP Atlas are subject to the limitation discussed in Section 4.5 and Section 6.2: the published literature from which they are drawn does not represent the full distribution of real-world formulation outcomes [65,100]. As noted under outlined priorities, prospective data deposition is the only change that would truly address this.

## 7. Conclusions

LNP research has made significant progress over the past decade, from demonstrating that lipid-based formulations are safe for intravenous use to enabling the delivery of billions of mRNA vaccine doses worldwide. This progress is real and should not be understated. However, a persistent problem remains: the field has developed a tendency to optimize formulations based on metrics that are easy to measure rather than on the biological outcomes that actually determine therapeutic efficacy.

This pattern appears in every area discussed in this review. Manufacturing bottlenecks, such as channel fouling and variability in microfluidic processes, are well known, yet they are rarely reported alongside the particle quality data they directly affect. ML models are trained on datasets biased toward successful outcomes, and no computational advance can overcome this without better input data. In microneedle delivery, it has never been directly tested whether the physical stresses of fabrication silently compromise the most critical property, that is the ability of a LNP to escape an endosome. Endosomal escape itself remains poorly understood and cannot be measured in a standardized way across laboratories. Additionally, the protein corona, which ultimately determines the biodistribution of LNPs, is almost always characterized using plasma from healthy volunteers, even when the intended patients are very different.

None of these issues require entirely new technology to solve. The necessary tools, platforms, and imaging methods already exist. What the field needs is consensus on which questions to prioritize, discipline to report unsuccessful experiments, and commitment to treat biological validation as a requirement rather than an afterthought. The seven priorities proposed here are a starting point for that change.

## Figures and Tables

**Table 1:** Chronological evolution of lipid-based nanocarriers from parenteral nutrition to gene therapy.

Decade	Key Development	Representative Product or Technology	Impact on the Field	Refs.
1960–1975	First intravenous lipid emulsions for total parenteral nutrition	Intralipid	First lipid emulsion approved for clinical intravenous use	[1]
1980–1990s	First drug-loaded parenteral lipid emulsions	Diazemuls (diazepam), Diprivan (propofol)	First drug-loaded lipid emulsion approved for clinical intravenous use	[1]
2000s	PEGylation strategies for NEs	PEGylated NEs with extended circulation and decreased protein binding	Reduced protein corona formation and prolonged systemic circulation	[4,10]
2010s	Ionizable cationic lipids	Research LNPs for siRNA (DLin-MC3-DMA)	pH-dependent charge switching for nucleic acid encapsulation and endosomal release	[5,9]
2018	First siRNA LNP clinical approval	Onpattro (patisiran; Alnylam Pharmaceuticals) (Cambridge, MA, USA)	First approved siRNA therapy using an ionizable LNP platform	[5]
2020–2021	First mRNA LNP clinical approvals	Comirnaty (Pfizer/BioN-Tech) (Pfizer, Andover, MA, USA; BioNTech, Mainz, Germany) and Spikevax (Moderna) (Cambridge, MA, USA) for COVID-19	First approved mRNA therapeutics of any kind	[6]
2022–present	Next-generation ionizable lipids; extrahepatic targeting strategies	SORT nanoparticles; ionizable cholesterol lipids (Tc-LNP)	Extrahepatic mRNA delivery, without ligand conjugation	[11,12]

PEG: polyethylene glycol; NE: nanoemulsion; LNP: lipid nanoparticle; siRNA: small interfering ribonucleic acid (RNA); mRNA: messenger RNA; COVID-19: coronavirus disease 2019; SORT: selective organ targeting; Tc-LNP: three-component LNP.

**Table 2:** Key manufacturing challenges in LNP production by microfluidics, their causes, proposed solutions, and relevant references.

Problem	Underlying Cause	Proposed Solution	Refs.
Microchannel fouling and clogging	Lipid–RNA–ethanol–water mixture adsorption onto PDMS/glass surfaces; clogging within 10–20 min; standard antifouling coatings’ deterioration in ethanol	TLP omniphobic coating (SCALAR-AF-chip); continuous operation > 3 h without fouling, EE > 85% maintained	[26]
Non-reproducible particle properties across device geometries	T-junction, SHM, and hydrodynamic flow -focusing production of LNPs of different sizes and EE under otherwise identical conditions	Critical influence of TFR and FRR on LNP size and PDI; cross-laboratory reproducibility requires reporting them	[21]
Limited throughput of single microfluidic channels	Single SHM channel capacity insufficient for clinical/commercial scale	Parallelization: 128 SHMs on a single chip, demonstrating scale-invariant LNP production	[27]
Residual ethanol incompatible with injection	Post-mixing suspension composition: ∼25% (*v*/*v*) ethanol, destabilizing particles over time	Downstream buffer exchange (tangential flow filtration or dialysis); careful process control to avoid particle damage	[17]
Thermal instability of liquid mRNA-LNP formulations	Water drives mRNA backbone hydrolysis, while freeze–thaw cycles cause aggregation and cargo leakage	Lyophilization with lyoprotectants (sucrose/trehalose); 20% *w*/*v* concentration required for full protection across three freeze–thaw cycles	[30,32,33]
Lyophilization protocols not transferable between formulations	Changes in lipid composition alter freeze-drying stress response; cycle must be reoptimized empirically for each new ionizable lipid	Formulation-specific re-optimization of cryoprotectant type, concentration, and drying cycle; universal protocol development	[32,33]

LNP: lipid nanoparticle; RNA: ribonucleic acid; PDMS: polydimethylsiloxane; TLP: tethered liquid perfluorocarbon; EE: encapsulation efficiency; SHM: staggered herringbone mixer; TFR: total flow rate; FRR: flow rate ratio; PDI: polydispersity index; mRNA: messenger RNA.

**Table 3:** Key barriers to clinical translation of LNP-loaded dissolvable microneedle platforms.

Barrier Category	Specific Limitation	Consequence for Clinical Translation	Refs.
Physicochemical validation	Reliance on DLS (size, PDI) and EE as surrogate markers for LNP integrity	Standard metrics do not assess endosomal escape capacity or confirm intactness of therapeutically active mRNA after microneedle fabrication	[43,44]
Mechanical testing standardization	Different in vitro test configurations yield non-comparable results	The lack of harmonized protocols prevents direct comparison of mechanical performance across studies and hinders the establishment of quality specifications	[41]
In vivo efficacy data	Published proof-of-concept studies based on healthy mouse models; disease-relevant models have not been reported	In vitro–in vivo correlations for this platform remain poorly understood; therapeutic efficacy in disease settings has not been demonstrated	[43,44]
Regulatory framework	Current FDA guidance on transdermal delivery systems does not address nucleic acid payloads or combination products containing LNPs	The lack of regulatory pathway creates uncertainty for product development and approval	[47]
Industrial scalability	LNP stability in microneedle matrices remains an unresolved industrial challenge	Technology is not yet ready for commercial-scale manufacturing; formulation and process re-optimization is required for each new LNP composition	[45]
Preclinical model relevance	Microneedle insertion dynamics differs significantly between mouse and human skin; more variable insertion efficacy in mouse skin	Dose estimates from mouse models carry uncertainty; human or porcine skin may be more predictive	[46]

LNP: lipid nanoparticle; DLS: dynamic light scattering; PDI: polydispersity index; EE: encapsulation efficiency; mRNA: messenger RNA; FDA: Food and Drug administration.

**Table 4:** Representative preclinical mRNA-LNP microneedle platforms reported between 2020 and 2026.

Nucleic Acid Cargo	LNP Formulation/Composition	Microneedle Type	Key Findings	Application	Ref.
Luciferase mRNA	Ionizable lipid: SM-102, DLin-MC3-DMA or ALC-0315; cholesterol or cholesterol analogue (sigmasterol, campesterol, beta-sitosterol, fucosterol); phospholipid: DSPE, DOPE, DOPS, DOTMA, or DOTAP; PEGylated lipid: DMG-PEG 2k, DSPE-PEG-COOH 2k, DSPE-PEG-COOH 5k, 18:0 PEG PE, or C16-PEG Ceramide	Dissolving MNP (PDMS mold, matrix: PVA, sucrose)	Increasing ionizable lipid content from 50% to 60% increased in vitro mRNA expression post manufacturing; SM-102 gave the highest expression among the three tested ionizable lipids, both before and after manufacturing; PVA outperformed the other tested polymer as the MN matrix	mRNA delivery platform	[43]
Luciferase encoding mRNA	Ionizable lipid: SM-102; cholesterol; phospholipid: DSPC; PEGylated lipid: DMG-PEG 2000	Dissolving MNP (PDMS mold, matrix: PVA, sucrose)	Lower manufacturing temperature (5 °C vs. 25 °C) better preserved mRNA-LNP function; ~87% activity loss occurred within 2 days at 40 °C, after which activity stabilized; PVA outperformed added sugars (sucrose, trehalose, glucose) as a stabilizing excipient	mRNA delivery platform	[44]
SARS-CoV-2 spike mRNA	Ionizable lipid: YK099; cholesterol; phospholipid: DSPC; PEG–lipid: DMG-PEG2000	Dissolvable bubble MNP (PDMS mold, PVA + trehalose matrix, PVP backing layer)	One-month stability at room temperature; rapid tip-backing separation; induction of a Th1-polarized T-cell response; lymph node targeting demonstrated in vivo	SARS-CoV-2 vaccine	[50]
SARS-CoV-2 mRNA	Ionizable lipid: SM-102; phospholipid: DSPC; cholesterol; PEG–lipid: DMG-PEG 2000; molar ratio 50:38.5:10:1.5	Powder-attached MNs (P-MAP): adhesive silicone-layered MNs (PDMS mold, PLA); mRNA-LNP lyophilized powder attached on adhesive silicon layer Coated MNs (C-MAP): liquid mRNA-LNP formulation coated onto PLA MN surfaces	Preservation of mRNA-LNP structural integrity after lyophilization/powder attachment; complete protection and viral clearance in mice immunized with the C-MAP counterpart; 6-month stability at 4 °C; reduced hepatic biodistribution vs. intramuscular injection	SARS-CoV-2 vaccine	[52]
SARS-CoV-2 spike mRNA	Ionizable lipid: cKK-E12 or Lipid 5; phospholipid: DOPE; cholesterol; PEG–lipid: C14-PEG2000 mRNA-LNP (molar ratio: 35:16:46.5:2.5 for cKK-E12/DOPE/cholesterol/C14-PEG2000; 38.4:12.3:47.4:1.9 for Lipid 5/DOPE/cholesterol/C14-PEG2000)	Dissolvable MNP (PDMS mold, dissolvable polymer: PVP, PVA or PVP:PVA blend)	Automated, standalone fabrication process by MN vaccine printer; more than 6-month shelf stability at room temperature; immune response in mice comparable to intramuscular injection	COVID-19 mRNA vaccine	[54]
eGFP encoding mRNA	Ionizable lipid: DLin-MC3-DMA; phospholipid: DOPE; cholesterol; PEG–lipid: DMG-PEG2000	Hydrogel-forming MNP (PDMS mold, crosslinked hydrogel-forming polymer matrix: Gantrez S-97, PEG 10 kDa, poly(acrylic acid) 450 kDa)	Spatially controlled post-manufacturing loading method; up to 140 µg payload delivered after six insertions; confirmed nanoparticle release and cellular uptake, with GFP transfection demonstrated in human skin ex vivo	Transdermal gene therapy	[55]

18:0 PEG PE: 1,2-distearoyl-sn-glycero-3-phosphoethanolamine-N-[methoxy(polyethylene glycol)]; ALC-0315: [(4-hydroxybutyl)azanediyl]di(hexane-6,1-diyl)bis(2-hexyldecanoate); C14-PEG2000: 1,2-dimyristoyl-sn-glycero-3-phosphoethanolamine-N-[methoxy(polyethylene glycol-2000)]; C16-PEG Ceramide: N-palmitoyl-sphingosine-1-[succinyl(methoxy(polyethylene glycol))]; cKK-E12: 3,6-bis(4-bis(2-hydroxydodecyl)amino)butyl)piperazine-2,5-dione; C-MAP: Coated Microneedle Array Patch; COVID-19: Coronavirus Disease 2019; DLin-MC3-DMA: (6Z,9Z,28Z,31Z-heptatriaconta-6,9,28,31-tetraen-19-yl-4-(dimethylamino)butanoate; DMG-PEG2000: 1,2-dimirystoyl-rac-glycero-3-methoxypolyethylene glycol-2000; DOPE: 1,2-dioleoyl-sn-glycero-3-phosphoethanolamine; DOPS: 1,2-dioleoyl-sn-glycero-3-phospho-L-serine; DOTAP: 1,2-dioleoyl-3-trimethylammonium propane; DOTMA: 1,2-di-O-octadecenyl-3-trimethylammonium propane; DSPC: 1,2-distearoyl-sn-glycero-3-phosphocholine; DSPE-PEG-COOH: 1,2-distearoyl-sn-glycero-phosphoethanolamine-N-[carboxy(polyethylene glycol)]; eGFP: enhanced Green Fluorescent Protein; Lipid 5: Heptadecane-9-yl 8-((2-hydroxyethyl)(8-(nonyloxy)-8-oxooctyl)amino)octanoate; LNP: Lipid Nanoparticle; MN: Microneedle; mRNA: Messenger Ribonucleic Acid; PEG: Polyethylene Glycol; PVA: Polyvinyl Alcohol; SARS-CoV-2: Severe Acute Respiratory Syndrome Coronavirus 2; SM-102: Ionizable lipid used in mRNA-LNP formulations; Th1: T-helper type 1 immune response; Th1-polarized: T-helper type 1 polarized immune response; YK099: 6-[[4-(decyloxy)-4-oxobutyl](2-hydroxyethyl)amino]-hexanoic acid, 2-octyldecyl ester.

**Table 5:** Representative clinical trials involving lipid nanoparticles for nucleic acid delivery, with particular emphasis on microneedle-based platforms [56].

Study Title	NCT Number	Status	Condition/ Disease	Intervention/Treatment	Sponsor	Phase
Establishing immunogenicity and safety of needle-free intradermal delivery of mRNA COVID-19 vaccine	NCT05315362	Unknown	Vaccination; infection; COVID-19	mRNA-1273 LNP vaccine (Spikevax, Moderna)—LNP encapsulated mRNA-based vaccine, administered through the intradermal route using a solid microneedle skin patch	Leiden University Medical Center (Leiden, The Netherlands)	2a
siRNA microneedle patches for ear keloid post-surgical scars	NCT07596628	Not yet recruiting	Keloid of ear lobe	siRNA microneedle patches—siRNA nanoplexes targeting SPARC mRNA embedded in the tips of hyaluronic acid dissolvable microneedles to enhance transcutaneous drug delivery *	Ong Kim Yao, National Skin Centre (Singapore)	2
Comparing the effect of siSPARC microneedle patch versus siSPARC + siLR4A microneedle patch on post-surgical scars	NCT06138964	Unknown	Keloid; wound heal; wound	siSPARC microneedle patch; siSPARC + siLR4A microneedle patch—short microneedles embedded with hydrolyzed RNA (siRNA) *	National Skin Centre (Singapore)	2
CDC-9 inactivating rotavirus vaccine (IRV) microneedle patch (MNP) in healthy adults	NCT06962904	Active, not recruiting	Rotavirus infections	CDC-9 inactivated rotavirus vaccine dissolving microneedle patch for intradermal administration *	Centers for Disease Control and Prevention (Atlanta, GA, USA)	1
Measles and rubella vaccine microneedle patch phase 1–2 age de-escalation trial	NCT04394689	Completed	Measles; rubella; vaccination; healthy	Measles rubella vaccine, delivered intradermally as a dissolving microneedle patch *	Micron Biomedical, Inc. (Atlanta, GA, USA)	1/2
Phase 1 evaluation of H1 influenza vaccine delivered by MIMIX MAP	NCT06125717	Completed	Influenza	H1 influenza vaccine delivered by VX-103 (a MIMIX MAP system)—microneedle platform with dissolving tips containing H1 influenza antigen and stabilizing excipient silk fibroin *	Terrestrial Bio, Inc. (Woburn, MA, USA)	1
A study to evaluate the safety and immunogenicity of two doses of a novel H5 central antigen mRNA-LNP in healthy adults	NCT07019883	Completed	Avian influenza	Influenza A/H5 antigenically central (AC)-Anhui RNA vaccine—nucleoside modified mRNA vaccine; the 2119-nt mRNA encapsulated in LNPs for intramuscular delivery	National Institute of Allergy and Infectious Diseases (NIAID) (Rockville, MD, USA)	1
Safety and immune responses after vaccination with an investigational RNA-based vaccine against malaria	NCT05581641	Completed	Malaria	BNT165b1 vaccine—RNA-LNP vaccine administered as intramuscular injection	BioNTech SE (Mainz, Germany)	1
A study to evaluate safety, tolerability, & immunogenicity of multiple formulations of BNT162b2 against COVID-19 in healthy adults	NCT04816669	Completed	SARS-CoV-2 infection; COVID-19	BNT162b2 RNA-based COVID-19 vaccine—frozen-liquid LNP formulation of BNT162b2 (a nucleoside-modified mRNA encoding the trimerized SARS-CoV-2 spike glycoprotein) with LNP size at the upper end of specification; intramuscular injection	BioNTech SE (Mainz, Germany)	3
Safety, tolerability, and pharmacokinetics of ARCT-032 in healthy adult subjects and adults with cystic fibrosis	NCT05712538	Completed	Cystic fibrosis	ARCT-032—mRNA coding for cystic fibrosis transmembrane conductance regulator protein formulated in a LNP, administered via nebulizer	Arcturus Therapeutics, Inc. (San Diego, CA, USA)	1
Safety and immunogenicity study of three mRNAs encoding HIV immunogens in adult participants without HIV and in overall good health in South Africa	NCT06694753	Active, not recruiting	HIV infections	mRNA-1645-eODGT8, mRNA-1645-CoreG28v2 and mRNA-1645-N332GT5 vaccines—HIV immunogens (eOD-GT8 60mer, core-g28v2 60mer, N332-GT5 gp151) delivered using an mRNA-LNP platform, administered by intramuscular injection	International AIDS Vaccine Initiative (New York, NY, USA)	1
COVID-19 variant immunologic landscape trial (COVAIL trial)	NCT05289037	Completed	COVID-19	mRNA-1273, mRNA-1273.351, mRNA-1273.529, mRNA-1273.617.2, BNT162b2 (B.1.1.529), BNT162b2 (B.1.1.351) vaccine candidates—LNP dispersion containing mRNA administered through intramuscular injection	National Institute of Allergy and Infectious Diseases (NIAID) (Rockville, MD, USA)	2
Safety and immunogenicity of chimeric hemagglutinin mRNA vaccine candidates	NCT07703514	Not yet recruiting	Influenza	Influenza A group 1 and influenza A group 2 mRNA chimeric hemagglutinin vaccine candidates (cH15/3 and cH4/3)—RNA encapsulated in LNPs for intramuscular delivery; LNP comprised ionizable lipid (ALC-0315), DSPC, cholesterol and PEG–lipid (ALC-0159)	National Institute of Allergy and Infectious Diseases (NIAID) (Rockville, MD, USA)	1
Efficacy study of COVID-19 mRNA vaccine in regions with SARS-CoV-2 variants of concern (CoVPN3008)	NCT05168813	Completed	SARS-CoV-2 infection; HIV infections; COVID-19	Moderna mRNA-1273 and mRNA-1273.222 COVID-19 vaccines—LNP dispersion of mRNA encoding the prefusion stabilized S protein of SARS-CoV-2 (mRNA-1273) and of the Omicron subvariants BA.4/.5 (mRNA-1273.222), formulated in LNPs containing four lipids (proprietary ionizable lipid SM-102, cholesterol, DSPC, PEG2000 DMG)	COVID-19 Prevention Network (Seattle, WA, USA)	2, 3
Study of circular RNA treatment in patients with radiation-induced xerostomia-1 (SPRINX-1)	NCT06714253	Recruiting	Radiation-induced xerostomia and hyposalivation	RXRG001—circular RNA encoding human aquaporin-1 encapsulated in LNP; intraductal administration to parotid gland(s)	RiboX Therapeutics Ltd. (Shanghai, China)	1, 2
Phase 1 study of a self-amplifying RNA vaccine (ITI-5000) in stage 2–3 triple negative breast cancer	NCT07652242	Recruiting	Triple negative breast cancer	ITI-5000 mRNA vaccine—self-amplifying RNA vaccine delivered inside LNP as an intramuscular injection	Immunomic Therapeutics, Inc. (Rockville, MD, USA)	1
IMP3-saRNA vaccine in advanced NSCLC	NCT07561723	Recruiting	Non-small cell lung cancer	IMP3-saRNA (YMN-136) vaccine—IMP3 self-amplifying RNA formulated with LNPs for intramuscular injection	West China Hospital (Chengdu, China)	1
Phase 1/2 study of intratumoral injection of STX-001 in advanced solid tumors as monotherapy or in combination with pembrolizumab	NCT06249068	Recruiting	Advanced solid tumor	STX-001—self-replicating RNA encoding IL-12, formulated in LNP for intratumoral injection	Strand Therapeutics Inc. (Boston, MA, USA)	1, 2

Data were manually retrieved from the ClinicalTrials.gov database [56] and analyzed through a detailed examination of each study title, summary and description. Studies were selected to illustrate microneedle-based delivery of nucleic acids/vaccines, as well as clinically relevant applications of LNP-formulated nucleic acids. The search strategy included the following terms: [(nucleic acid OR RNA) AND (lipid nanoparticle OR LNP)] (107 studies); [(nucleic acid OR RNA) AND (lipid nanoparticle OR LNP) AND (local delivery)] (14 studies), [(nucleic acid OR RNA) AND (lipid nanoparticle OR LNP) AND microneedle] (1 study), [(nucleic acid OR RNA) AND microneedle] (8 studies); study start: 1 January 2020–31 July 2026 (accession on 1 August 2026). * Not reported to be LNP-microneedle platforms. mRNA: messenger ribonucleic acid; LNP: lipid nanoparticle; siRNA: small interfering RNA; SPARC: secreted protein acidic and cysteine-rich; siSPARC: small interfering RNA against SPARC; siSPARC + siLR4A: small interfering RNA against SPARC and IL4-RA; MAP: microneedle array patch; DSPC: distearoylphosphatidylcholine.

**Table 6:** Representative applications of DoE, ML, and hybrid approaches in LNP development.

Application/Cargo	Methodology	Key Findings/Optimization Target	Validation Approach	Ref.
LNP for cellular tropism	DoE + High-throughput Screening + ML	Screening of 180 LNP formulations with diverse lipid chemistries; identification of formulations preferentially targets immune cells while reducing hepatic uptake; in vivo validation in mice	In vivo confirmation	[60]
mRNA-LNP vaccines	DoE + ML (XGBoost, Random Forest, SVM, k-NN, Lasso, ANN) + ANN-DoE hybrid	Evaluation of ionizable lipid type, ionizable lipid-to-cholesterol ratio, N/P ratio, FRR, and TFR against PDI, ZP, and EE; ANN performance of R^2^ = 0.73–0.999; outperformance of individual ML models by the ANN-DoE hybrid	Internal train/test split	[67]
mRNA (ionizable lipid discovery)	Combinatorial chemistry (584-lipid library) + ML-guided virtual screening (40,000-lipid library)	Generation of a chemically diverse 584-lipid combinatorial library; ML-guided screening of a 40,000-lipid virtual library with synthesis and testing of the top 16 candidates; identification of lipid 119–23, which outperforms benchmark ionizable lipids (MC3, SM-102) in muscle and immune cell transfection across several tissues	Internal train/test split	[71]
mRNA delivery (in vitro, in vivo)	CFD simulation + microfluidic HFF device + ML modeling	Size control of LNPs (30–270 nm) via flow rate; establishment of an LNP self-assembly constant; higher in vitro uptake and transfection for smaller LNPs; size-dependent biodistribution in vivo	In vivo confirmation	[72]
Fetal gene delivery (siRNA)	DoE + ML (Random Forest, XGBoost, SVR, Lasso, LightGBM) + SHAP analysis	Screening of a 41-formulation library; identification of ZP, particle size, and PEG–lipid properties as top predictors of transplacental transport; 622% increase in transport achieved through ML-guided optimization	Internal train/test split	[73]
mRNA-LNP (postencapsulation process)	Microfluidic full-factorial DoE (main lipid type, sterol type, FRR, chip design) + XGBoost ML, refined by semisupervised learning	Effects of chip design and lipid composition on preformed-vesicle size and PDI, with smaller vesicles produced by higher-mixing-efficiency chips; consistent size increase and PDI decrease after postencapsulation relative to preformed vesicles. XGBoost-based prediction of size and EE improved through semisupervised learning	Internal train/test split	[74]
Plasmid DNA (pDNA), 6 cell types	ML (SHAP analysis) on HTS-generated pDNA-LNP transfection datasets	Prediction errors of 5–10% depending on cell type; consistent composition parameters identified across cell types (helper lipid molar % of charged species 9–50%; cationic/zwitterionic helper lipids); additional modulators of cell type-preferentiality	Internal train/test split	[75]
siRNA	QSAR (ANN, SVM, PLS) with evolutionary-algorithm descriptor selection—purely in silico, no wet-lab DoE	Prediction of log(siRNA dose) with R^2^val = 0.86–0.89 (validation set 1) and 0.75–0.80 (validation set 2); best performance from ANN; R^2^val improved from 0.47 to 0.96 after restricting the training dataset to the applicability domain	Held-out test set	[76]

ANN: Artificial Neural Network; CFD: Computational Fluid Dynamics; DoE: Design of Experiments; EE: encapsulation efficiency; FRR: flow rate ratio; HFF: hydrodynamic flow focusing; k-NN: k-Nearest Neighbors; Lasso: Least absolute shrinkage and selection operator; LightGBM: Light Gradient Boosting Machine; ML: machine learning; N/P: nitrogen-to-phosphate ratio; PDI: polydispersity index; PLS: Partial Least Squares; QSAR: Quantitative Structure–Activity Relationship; SHAP: SHapley Additive exPlanations; SVM: Support Vector Machine; SVR: Support Vector Regression; TFR: total flow rate; XGBoost: eXtreme Gradient Boosting; ZP: zeta potential.

**Table 7:** Current status and remaining evidence gap for the cross-cutting challenges identified in this review.

Problem Area	Status	Authors’ Assessment
Channel fouling	Mitigated under the tested conditions; scale validation required	The TLP coating substantially mitigated component deposition under the tested conditions; long duration and GMP-scale validation remain required.
Lyophilization transferability	Unresolved, practically urgent	Each new lipid composition generally requires formulation-specific freeze-drying optimization, whereas high encapsulation efficiency and particle-size control are routinely achievable in many systems.
Endosomal escape post-microneedle fabrication	Potentially compromised, not directly tested	Fabrication stresses may also affect escape function, but direct before-versus-after functional testing is lacking.
Endosomal escape ceiling (2–10%)	Consistently low in the systems examined, generality unresolved	Distinct assays report low escape values, but their direct comparability and the generality of this range remain unresolved.
Machine learning-guided lipid screening (e.g., AGILE)	Promising, currently overstated	The speed of in silico candidate generation does not indicate prediction reliability when the underlying training data are from unpurified lipid batches.
SORT mechanistic basis	Ambiguous	It is unclear from the published literature whether the serum protein-binding mechanism was predicted before screening or reconstructed afterward to fit the result.

TLP: tethered liquid perfluorocarbon; GMP: good manufacturing practice; SORT: selective organ targeting.

## Data Availability

No new data were created or analyzed in this study. Data sharing is not applicable to this article.

## Abbreviations

The following abbreviations are used in this manuscript:

**Abbreviation**	**Full Term**
18:0 PEG PE	1,2-distearoyl-sn-glycero-3-phosphoethanolamine-N-[methoxy(polyethylene glycol)
AI	Artificial intelligence
ALC-0315	[(4-hydroxybutyl)azanediyl]di(hexane-6,1-diyl)bis(2-hexyldecanoate)
ANN	Artificial Neural Network
ApoE	Apolipoprotein E
API	Application programming interface
C14-PEG2000	1,2-dimyristoyl-sn-glycero-3-phosphoethanolamine-N-[methoxy(polyethylene glycol-2000)]
C16-PEG Ceramide	N-palmitoyl-sphingosine-1-[succinyl(methoxy(polyethylene glycol))];
CFD	Computational Fluid Dynamics
cKK-E12	3,6-bis(4-bis(2-hydroxydodecyl)amino)butyl)piperazine-2,5-dione
C-MAP	Coated Microneedle Array Patch
CNS	Central nervous system
COVID-19	Coronavirus disease 2019
CQA	Critical quality attribute
Cryo-TEM	Cryogenic transmission electron microscopy
DLS	Dynamic light scattering
DMG-PEG2000	1,2-dimirystoyl-rac-glycero-3-methoxypolyethylene glycol-2000
DLin-MC3-DMA	(6Z,9Z,28Z,31Z)-Heptatriaconta-6,9,28,31-tetraen-19-yl 4-(dimethylamino)butanoate
DoE	Design of Experiments
DOPE	1,2-Dioleoyl-sn-glycero-3-phosphoethanolamine
DOPC	1,2-Dioleoyl-sn-glycero-3-phosphocholine
DOPS	1,2-dioleoyl-sn-glycero-3-phospho-L-serine
DOTAP	1,2-dioleoyl-3-trimethylammonium propane
DOTMA	1,2-di-O-octadecenyl-3-trimethylammonium propane; DSPC: 1,2-distearoyl-sn-glycero-3-phosphocholine
DSPC	1,2-Distearoyl-sn-glycero-3-phosphocholine
DSPC-PEG-COOH	1,2-distearoyl-sn-glycero-phosphoethanolamine-N-[carboxy(polyethylene glycol)]
EE	Encapsulation efficiency
EEA1	Early endosome antigen 1
eGFP	enhanced Green Fluorescent Protein
ESCRT	Endosomal sorting complexes required for transport
FDA	Food and Drug Administration
FRR	Flow rate ratio
GFP	Green Fluorescent Protein
GMP	Good Manufacturing Practice
HFF	Hydrodynamic Flow Focusing
HTS	High-Throughput Screening
IM	Intramuscular
k-NN	k-Nearest Neighbors
LAMP1	Lysosomal-associated membrane protein 1
LANCE	Large-scale assembled nanoparticle characterization experiment
Lasso	Least Absolute Shrinkage and Selection Operator
LDL	Low-density lipoprotein
LightGBM	Light Gradient Boosting Machine
Lipid 5	Heptadecane-9-yl 8-((2-hydroxyethyl)(8-(nonyloxy)-8-oxooctyl)amino)octanoate
LNP	Lipid nanoparticle
LNPDB	Lipid nanoparticle database
MAP	Microneedle Array Patch
ML	Machine learning
MN	Microneedle
MNP	Microneedle Patch
mRNA	Messenger RNA
N/P	Nitrogen-to-Phosphate Ratio
NE	Nanoemulsion
NIAID	National Institute of Allergy and Infectious Diseases
PDI	Polydispersity index
PDMS	Polydimethylsiloxane
PEG	Polyethylene glycol
PFD	Perfluorodecalin
pKa	Negative base-10 logarithm of the acid dissociation constant
PLS	Partial Least Squares
PVA	Polyvinyl Alcohol
QSAR	Quantitative Structure–Activity Relationship
R^2^	Coefficient of determination
Rab5	Ras-related protein 5
Rab7	Ras-related protein 7
RNA	Ribonucleic acid
SARS-CoV-2	Severe Acute Respiratory Syndrome Coronavirus 2
SHAP	SHapley Additive exPlanations
SHM	Staggered herringbone mixer
siRNA	Small interfering RNA
SMILES	Simplified molecular-input line-entry system
SM-102	Heptadecane-9-yl 8-(2-hydroxyethyl)(6-oxo-6-(undecyloxy)hexyl)amino)octanoate
SNARE	Soluble NSF attachment protein receptor
SORT	Selective organ targeting
SVEM	Self-validated ensemble model
SVM	Support Vector Machine
SVR	Support Vector Regression
Tc-LNP	Three-component lipid nanoparticle
TFR	Total flow rate
Th1	T-helper type 1 immune response
Th1-polarized	T-helper type 1 polarized immune response
TLP	Tethered liquid perfluorocarbon
TPN	Total parenteral nutrition
t-SNE	t-distributed stochastic neighbor embedding
UMAP	Uniform manifold approximation and projection
VBC	Vesicle budding and collapse
XGBoost	eXtreme Gradient Boosting
YK099	6-[[4-(decyloxy)-4-oxobutyl](2-hydroxyethyl)amino]-hexanoic acid, 2-octyldecyl ester
ZP	Zeta potential
